# Supplementary figures and images for: A chemical signal in human female tears lowers aggression in males
Source: PLoS Biol. 2023 Dec 21;21(12):e3002442. doi: 10.1371/journal.pbio.3002442 (PMC10734982; doi:10.1371/journal.pbio.3002442)

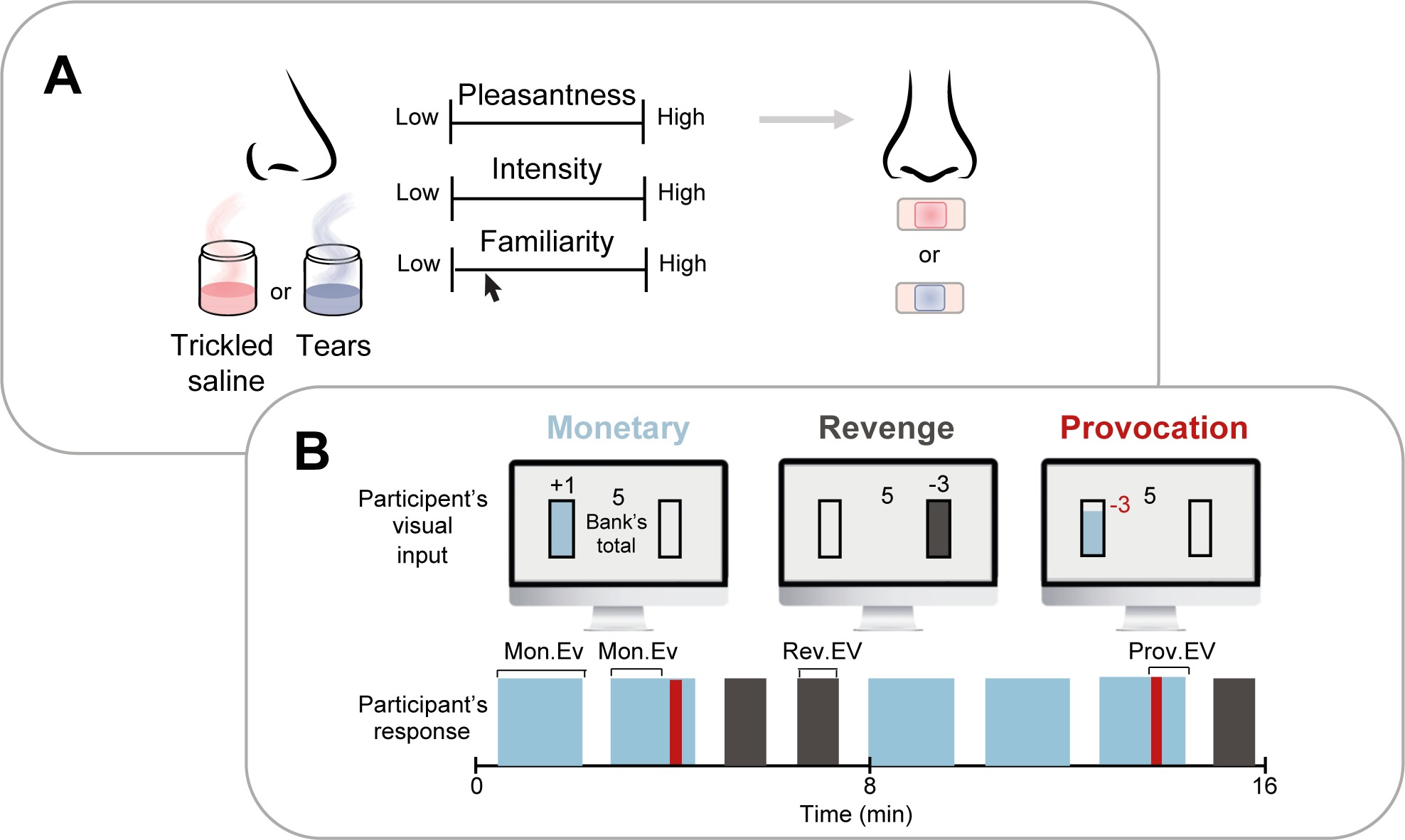

Supplement: S1 Fig — In a within-participant design, participants were exposed to trickled saline on one day, and tears on the other (counterbalanced for order and double-blind). (A) First, participants sniffed the stimulus (tears/saline) from a jar and rated odor perception 10 times. Next, a stimulus-impregnated pad was placed under the participant’s nostril for the rest of the experiment. Following that, participants engaged in the (B) PSAP game during which they could earn money for themselves (monetary response) or reduce money from their fictitious opponent at no personal gain (revenge response). During the game, they were provoked by money being taken from them by the fictitious opponent (provocation event). (TIF) [file pbio.3002442.s004.tif]

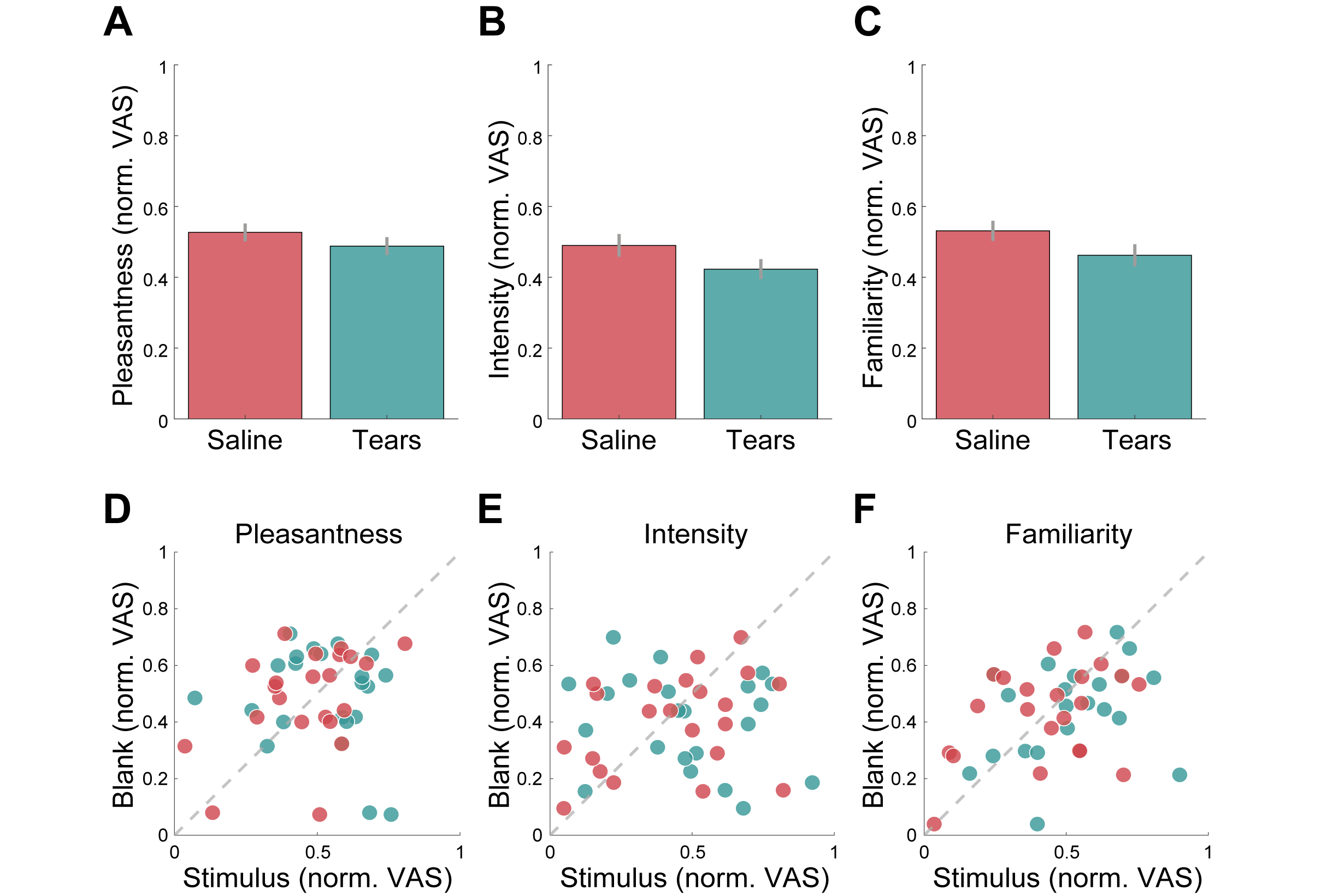

Supplement: S2 Fig — The bar plot depicts the mean ratings of (A) pleasantness, (B) intensity, and (C) familiarity of tears versus trickled saline. Whiskers represent the SE. The scatter plots depict of the normalized VAS ratings of saline solution (blank) and stimulus (tears in blue and trickled saline in red) for (D) pleasantness, (E) intensity, and (F) familiarity. Each dot represents the average of 6 blank sniffs and 10 stimulus sniffs (normalized to min-max values) of each participant. The data in (D-F) are presented along a unit slope line (X = Y), such that if points accumulate above the line, this implies higher values after tears; if points accumulate below the line, this implies higher values after saline; and if points are distributed around the line, this implies no difference. Data used to generate graphs can be found in S1 Data. (TIF) [file pbio.3002442.s005.tif]

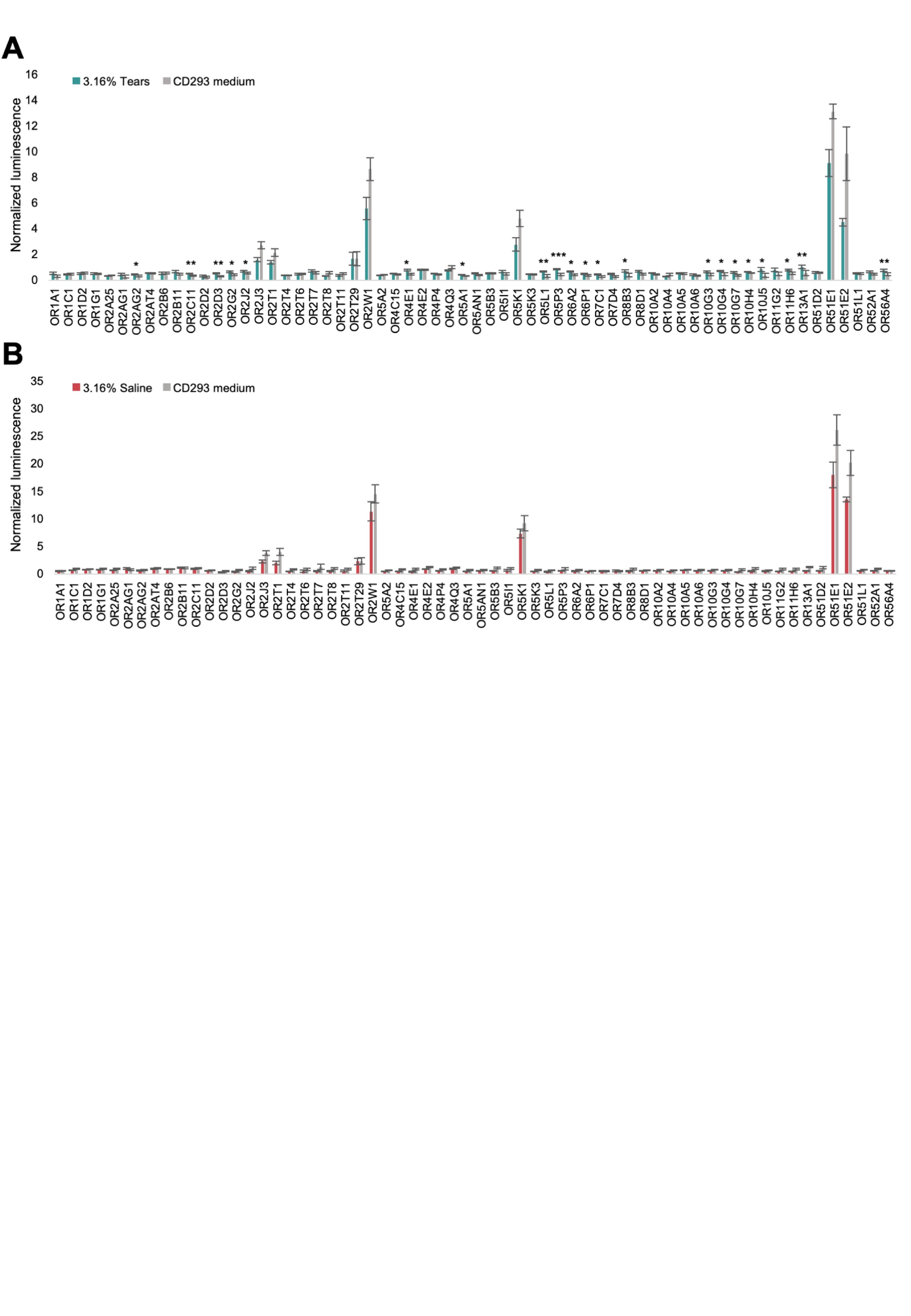

Supplement: S3 Fig — Screening for olfactory receptor (OR) activation in vitro by 3.16% (v/v) (A) tears (blue) and (B) trickled saline (red). The response of 62 human ORs was normalized to that of the empty vector (pCI). Bars represent the mean normalized luminescence (Luc/ Rluc), and error bars are standard error (SEM), n = 4. For screening only, the luminescence induced by tears/saline was compared to that of the solvent (CD293 medium, in gray) by one-tailed paired t tests. * = p < 0.05, ** = p < 0.01, *** = p < 0.001. Data used to generate graphs can be found in S2 Data. (TIF) [file pbio.3002442.s006.tif]

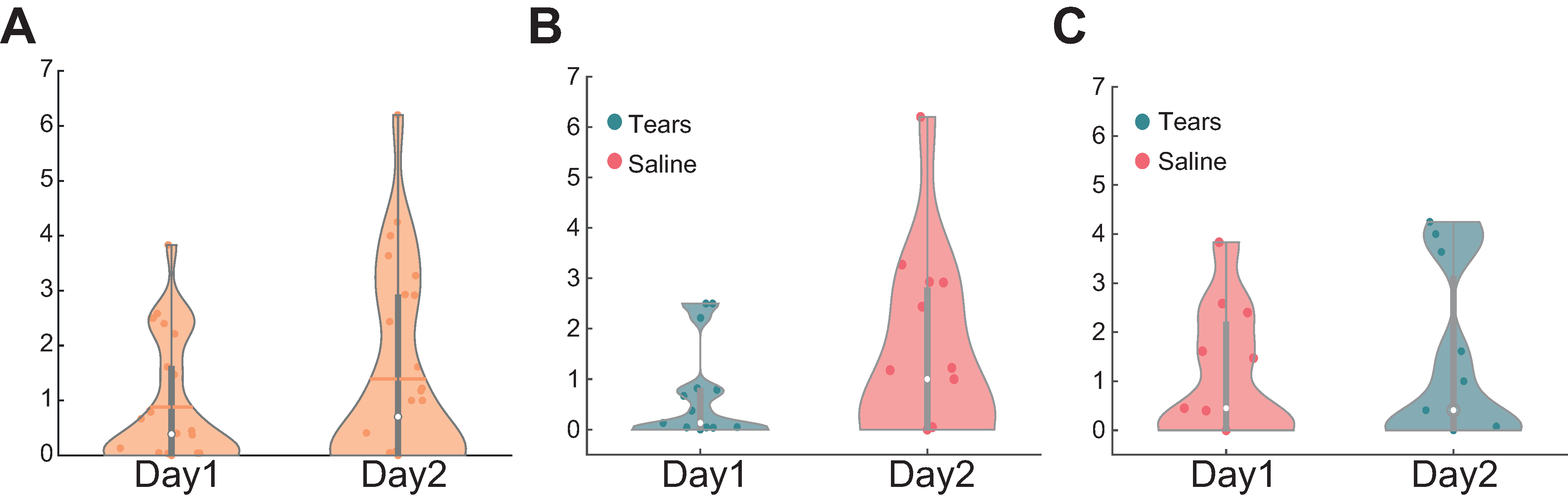

Supplement: S4 Fig — The behavioral effect of tears in the MRI was subtle, possibly since a day-after-day experiment inside the MR scanner rendered participants significantly more aggressive on the second day, regardless of condition (mean APR difference regardless of condition (day 2—day 1) = 0.511 ±1.2 APR, permutation p = 0.038, Mielke and Berry’s R = 0.125) as depicted in (A). Violin plot of APR score between days, regardless of condition. Thus, participants who sniffed tears on the first day exhibited a remarkable 73.6% lower aggression under tears (mean APR tears day 1 = 0.677 ± 0.942, mean APR saline day 2 = 1.413 ± 1.814, Shapiro–Wilk, W = 0.704, p < 0.001, implying a nonnormal distribution dictating a nonparametric test: Wilcoxon signed rank Z = 11, p = 0.017, effect size (rrb) = 0.758. If we nevertheless use the parametric approach, the effect remains the same: t (14) = 2.34, p = 0.034, Cohen’s d = 0.605), yet participants who sniffed saline on the first day had no difference in the levels of aggressiveness on the second day under tears (mean APR saline day 1 = 1.159 ± 1.332, mean APR tears day 2 = 1.362 ± 1.751, Wilcoxon signed rank Z = 16, p = 0.83, effect size (rrb) = 0.111. If we nevertheless use the parametric approach, the effect remains the same: t (10) = 0.579, p = 0.578, Cohen’s d = 0.175) as depicted in (B) and (C) respectively. (B) Violin plot of APR by day and by stimulus when day 1 was tears. (C) Violin plot of APR by day and by stimulus when day 1 was trickled saline. * = p < 0.05. Each dot in the violin plots (B-E) represents a participant. The white dot represents the median, and the gray bar represents the quartiles. Data used to generate graphs can be found in S1 Data. (TIF) [file pbio.3002442.s007.tif]

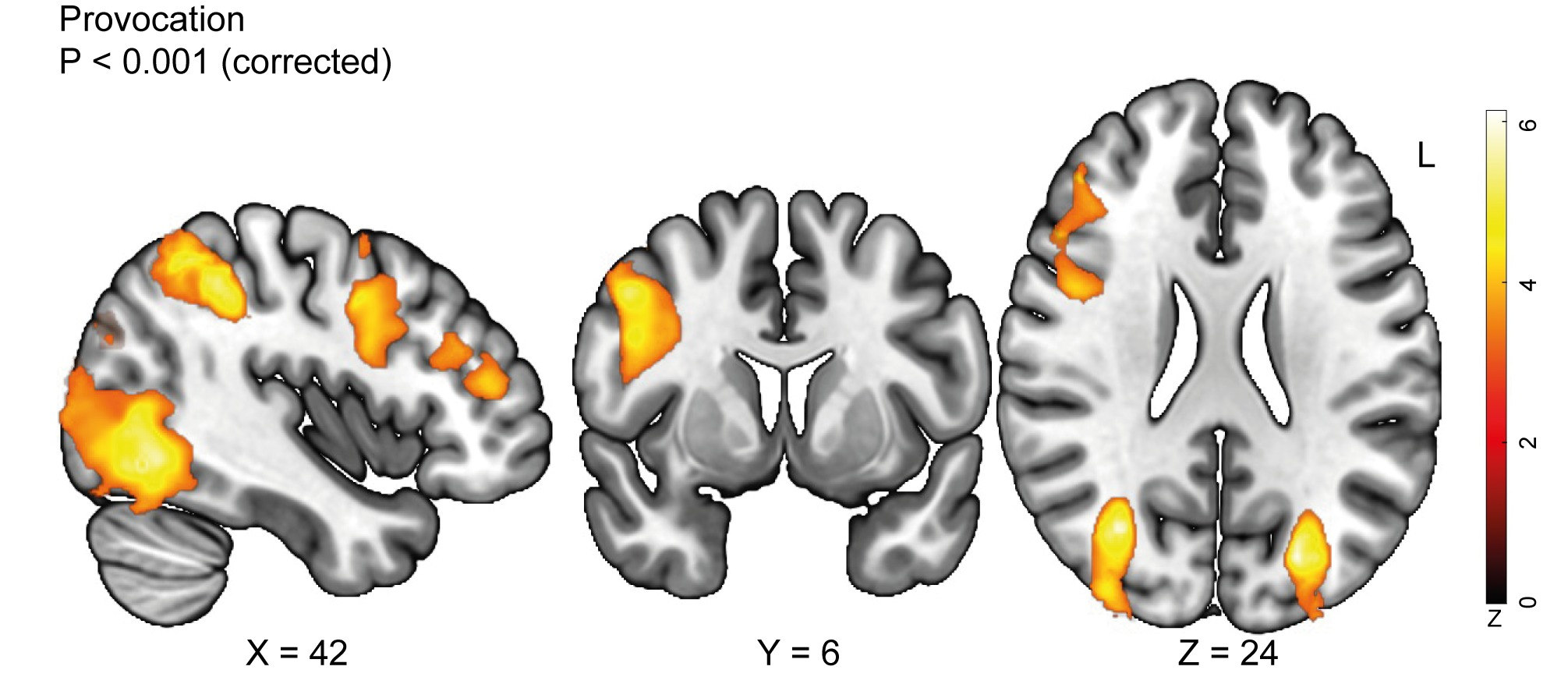

Supplement: S5 Fig — Statistical parametric map of the GLM provocation event, n = 24. The color bar represents z-values. P value is depicted. See full list of activated areas in S3 Table. Data are available at https://openneuro.org/datasets/ds004274. (TIF) [file pbio.3002442.s008.tif]

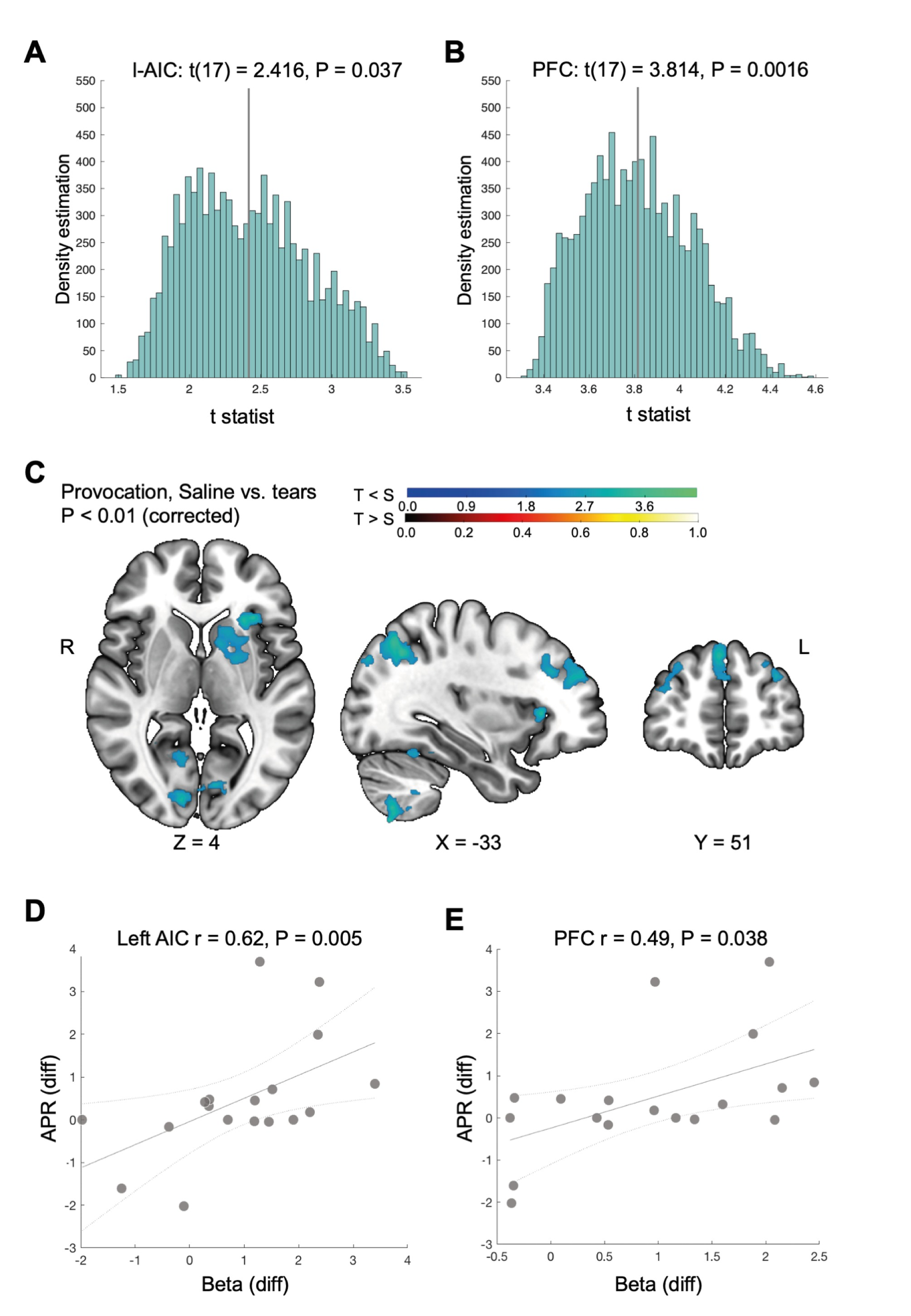

Supplement: S6 Fig — In the bootstrap analysis, participants were randomly selected 10,000 times to counterbalance for stimuli order. Beta values for (A) left AIC and (B) PFC were then compared for each stimulus. The histograms represent the distribution of the t statistic of all repetitions for each ROI. The gray horizontal line represents the mean statist. The mean t statists and the p-values are depicted. (C) Statistical map of the GLM ANOVA Provocation with an added level of saline vs. tears (tears < saline in blue; tears > saline in red), counterbalanced for order, n = 18. Z threshold > 2.31, cluster corrected to p = 0.05. Color bars represent z-values. Correlation between differences in behavioral APR scores (saline -tears) and differences in beta values (saline- tears) of (D) left AIC and (E) PFC. Each dot represents a participant. The continuous line represents the fit. The dashed line marks the confidence bounds. Spearman rank correlation coefficient and p-values are depicted. Data used to generate graphs can be found in S1 Data; fMRI data are available at https://openneuro.org/datasets/ds004274. (TIF) [file pbio.3002442.s009.tif]

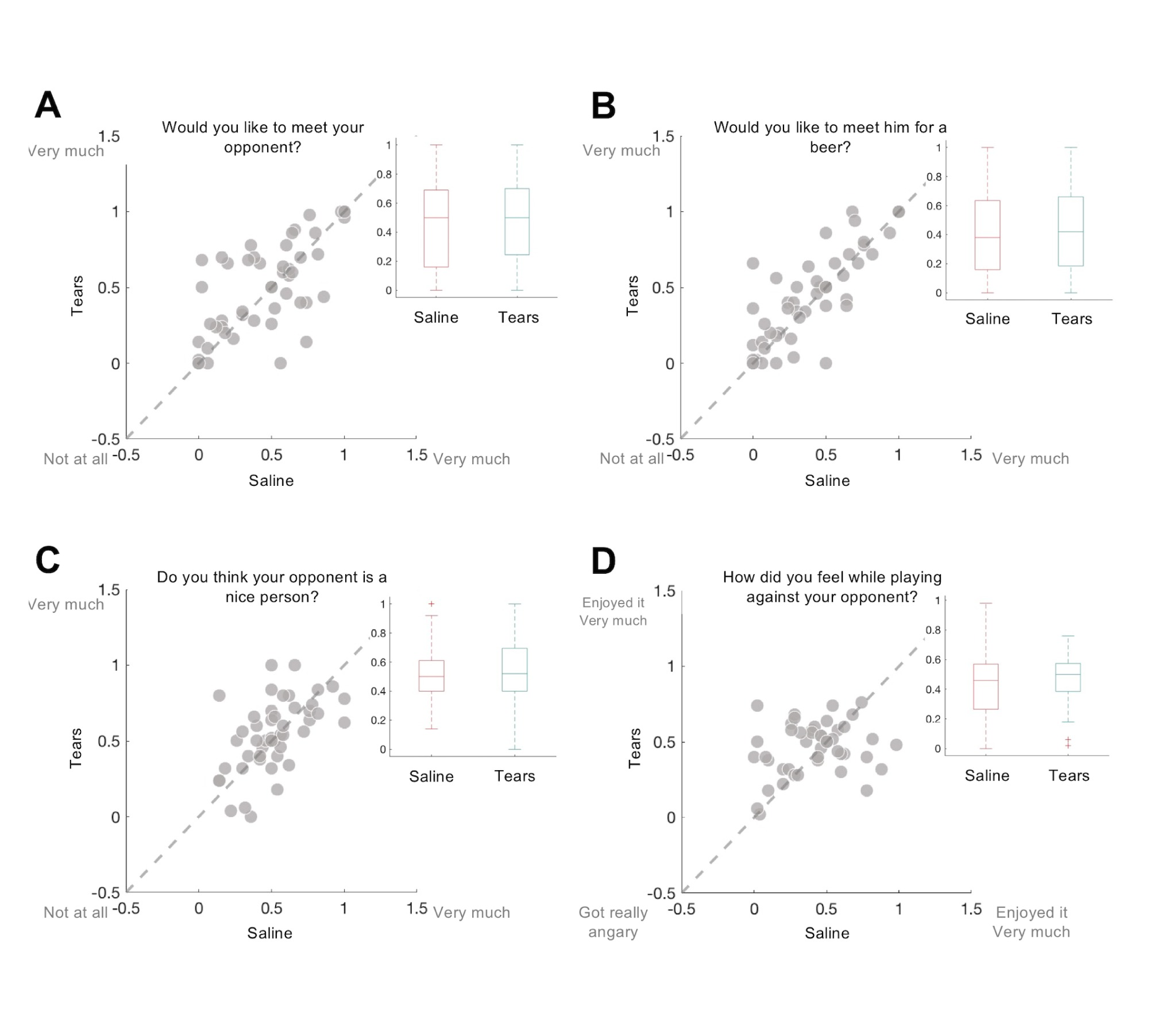

Supplement: S7 Fig — Participants completed a questionnaire that included social questions regarding their opponent at the end of each experimental day (i.e., once after sniffing tears and once after sniffing saline). Participants answered the following questions using a VAS ranging from “very much” to “not at all”: (A) Would you like to meet your opponent? (Shapiro–Wilk, W = 0.94, p < 0.03, implying a nonnormal distribution dictating a nonparametric test: Wilcoxon signed rank Z = 330, p = 0.195, corrected p = 0.78). (B) Would you like to meet him for beer? (Shapiro–Wilk, W = 0.92, p = 0.003, implying a nonnormal distribution dictating a nonparametric test: Wilcoxon signed rank Z = 293, p = 0.077, corrected p = 0.3). (C) Do you think your opponent is a nice person? (t(50) = −0.816, p = 0.42, corrected p > 0.99). (D) How did you feel while playing against your opponent? Participants answered this question using a VAS ranging from “enjoyed it very much” to “Got really angry” (Shapiro–Wilk, W = 0.944, p < 0.02, implying a nonnormal distribution dictating a nonparametric test: Wilcoxon signed rank Z = 284, p = 0.14, corrected p = 0.56). Participants did not show a difference in their social attitude toward their opponent across stimuli. Data used to generate graphs can be found in S1 Data. (TIF) [file pbio.3002442.s010.tif]

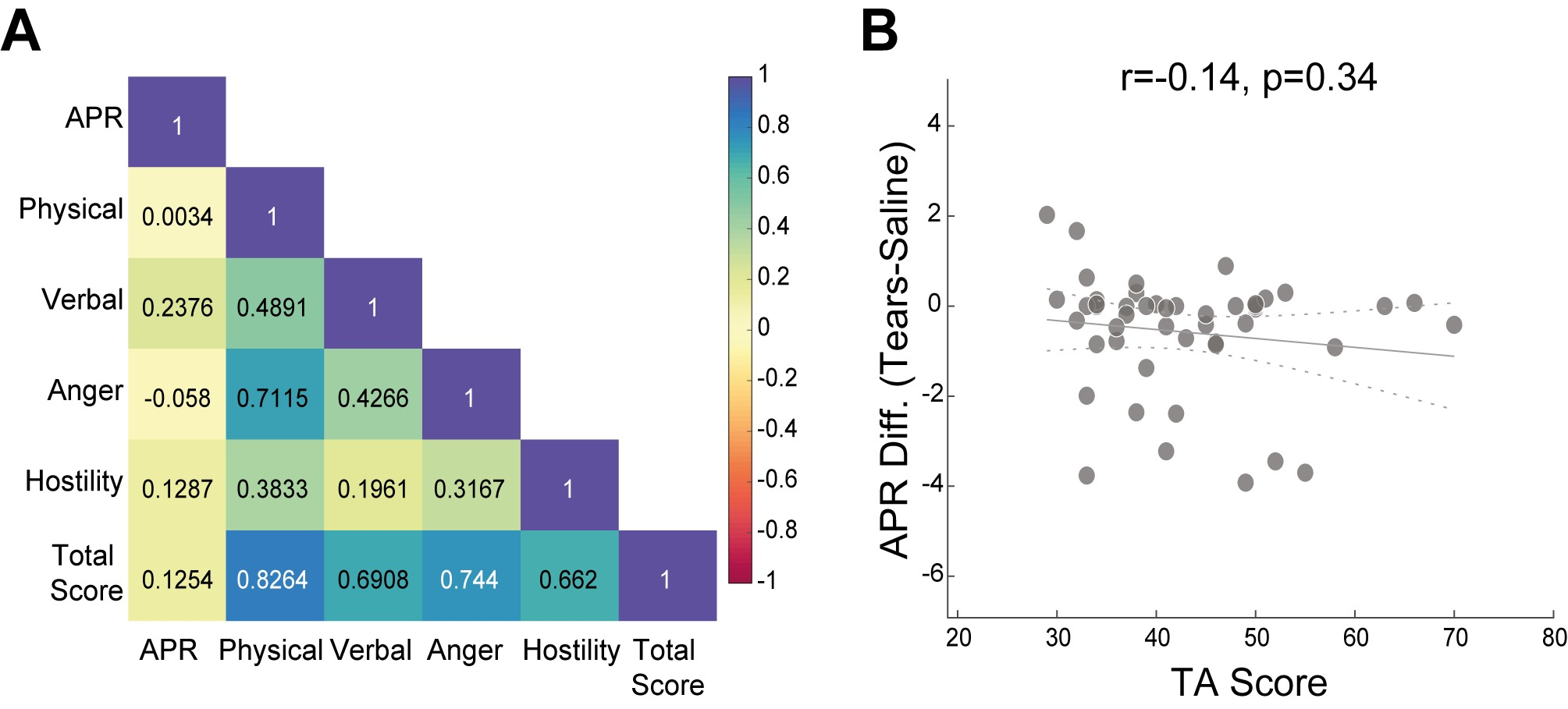

Supplement: S8 Fig — (A) Correlation matrix of AGQ; total score and the different factors with the APR difference between stimuli (Tears–Saline), n = 49. The color bar represents the Spearman rank correlation coefficient (r), also depicted. All p-values > 0.5 (corrected for multiple comparisons). (B) Correlation between STAI score and the APR difference between stimuli (Tears–Saline). Each dot represents a participant, n = 47 (2 participants did not completed this questionnaire). The continuous line represents the fit. The dashed line marks the confidence bounds. Spearman rank correlation coefficient and p-value are depicted. Data used to generate graphs can be found in S1 Data. (TIF) [file pbio.3002442.s011.tif]

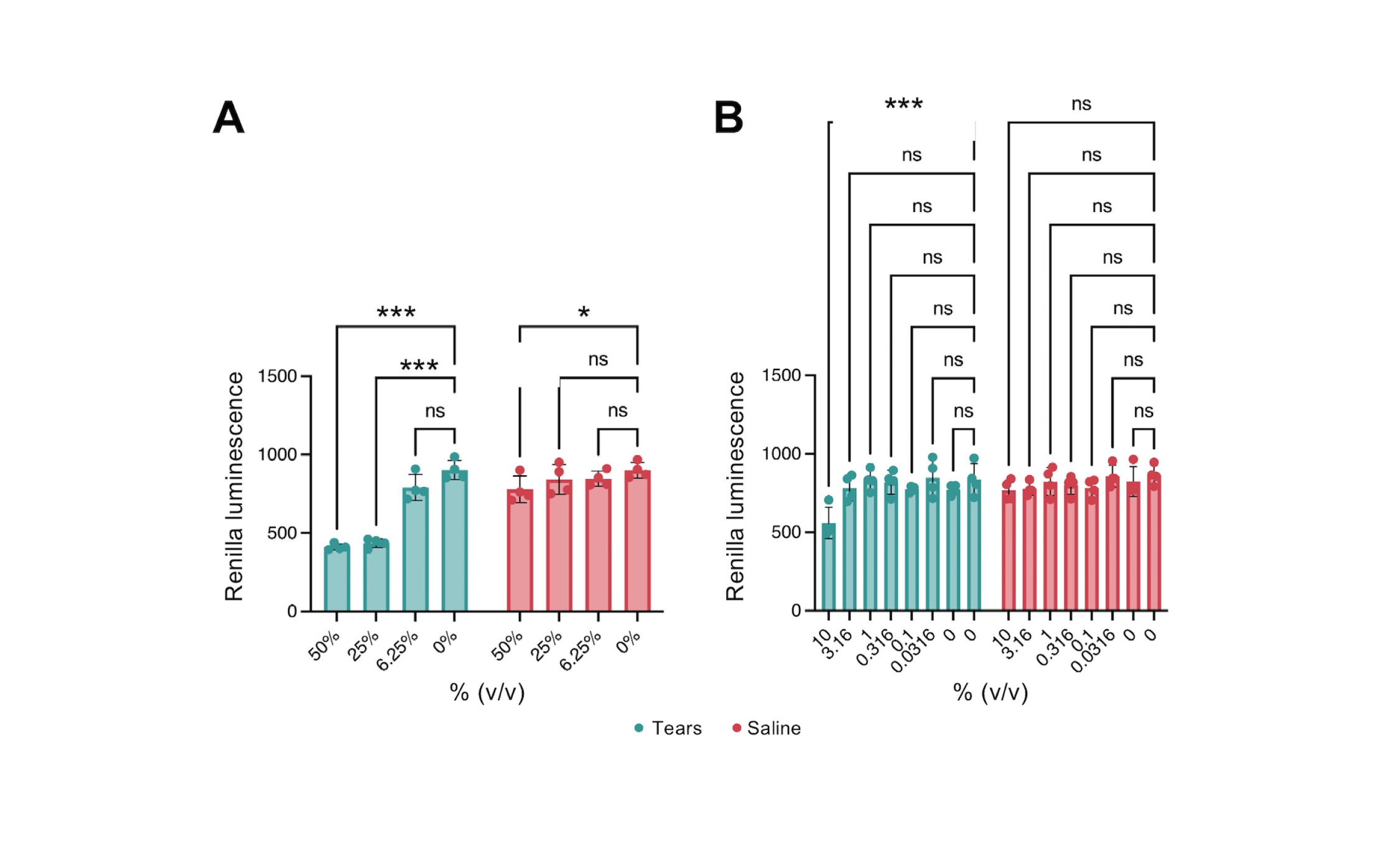

Supplement: S9 Fig — We monitored Renilla luciferase luminescence produced by cells (see Methods) transfected with the empty vector negative control (pCI), testing a wide range of stimuli concentrations, tears in blue and trickled saline in red. (A) 50%, 25%, and 6.25% (v/v in CD293 stimulation medium). Tears decreased the luminescence, indicating an OR-independent effect on cells at 50% and 25% (p < 0.001). At 6.25%, the nonspecific effect was close to significant with p = 0.0545. Therefore, we then refined the concentration range to (B) 10% to 0.0316% (v/v). The maximum tears concentration without nonspecific OR effects was 3.16%. Statistics are done with a multiple comparison 2-way ANOVA (Dunnett test, p > 0.05 = ns; 0.05 < p < 0.01 = *; 0.01 < p < 0.001 = **; p < 0.001 = ***). Data used to generate graphs can be found in S3 Data. (TIF) [file pbio.3002442.s012.tif]

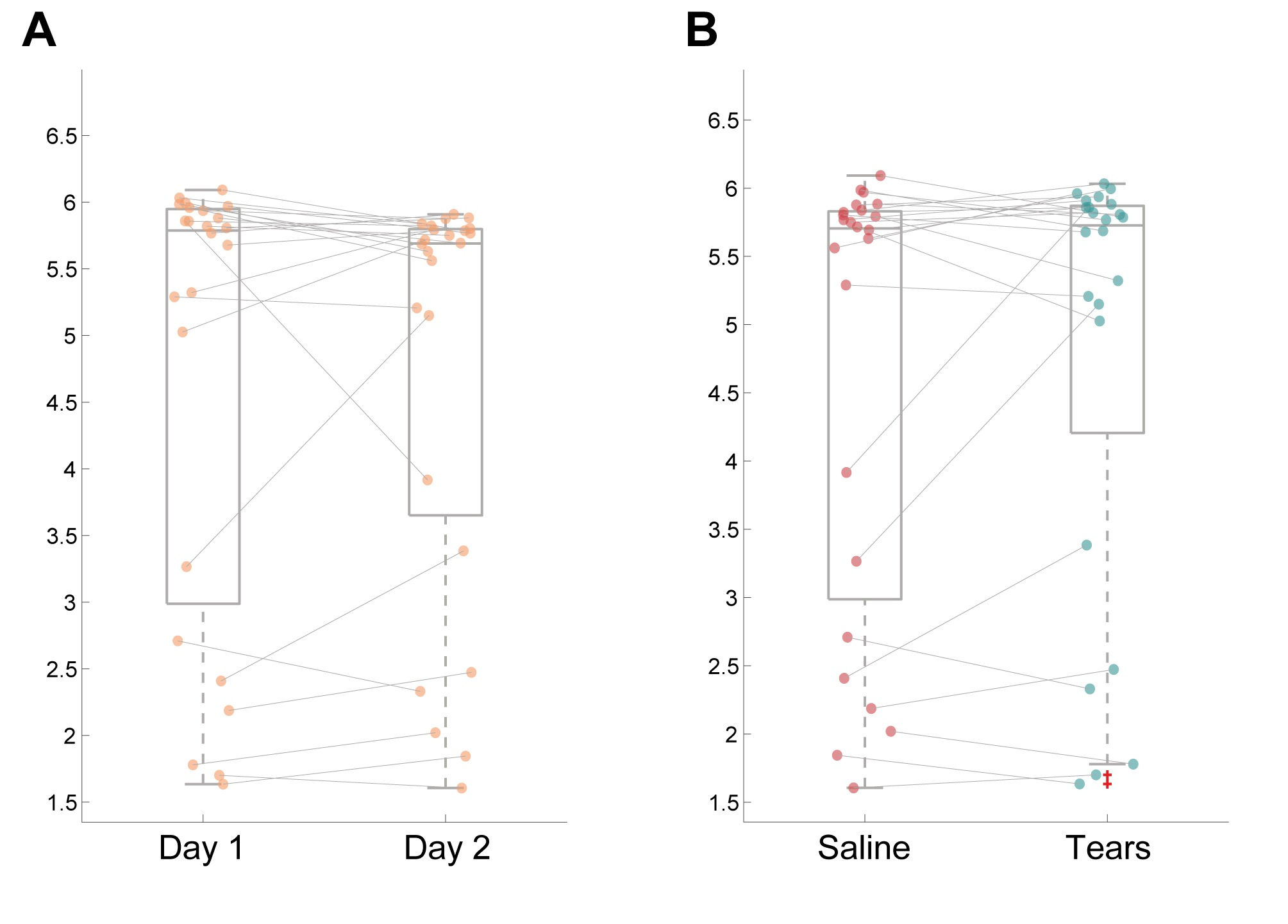

Supplement: S10 Fig — (A) No difference in provocation event length between days (Shapiro–Wilk, W = 0.817, p < 0.001, implying a nonnormal distribution dictating a nonparametric test: Wilcoxon signed rank Z = 156, p = 0.86, effect size (rrb) = 0.16). (B) No difference in provocation event length between stimuli, saline in red and tears in blue (Shapiro–Wilk, W = 0.78, p < 0.001, implying a nonnormal distribution dictating a nonparametric test: Wilcoxon signed rank Z = 142, p = 0.95, effect size (rrb) = 0.048). The rectangle reflects the upper and the lower interquartile (25th to the 75th percentiles), and the whiskers are minimum and maximum non-outlier (no more than 1.5 * IQR of the upper and lower hinges). Outlying points are plotted individually. The line inside the box is the sample median. Each point is a participant, and the line connects the repeated measure. Data used to generate graphs can be found in S1 Data. (TIF) [file pbio.3002442.s013.tif]
