# Supplementary material for: A chemical signal in human female tears lowers aggression in males
Source: PLoS Biol. 2023 Dec 21;21(12):e3002442. doi: 10.1371/journal.pbio.3002442 (PMC10734982; doi:10.1371/journal.pbio.3002442)
Supplement: S1 Table — The table summarizes the screening after OR activation in vitro, of 62 human ORs, induced by tears and trickled saline. The results are depicted in Fig 1. P values of one-tail paired t test comparison between solvent (CD293 medium) to 3.6% (v/v) tears or saline. Activ = considered as activated receptor, Inhib = considered as inhibited receptor. The corresponding p-values depicted in the table. (DOCX) [file pbio.3002442.s014.docx]

| OR subtype | OR response to tears | P-value  (OR response to tears) | OR response to saline | P-value  (OR response to saline) |
| --- | --- | --- | --- | --- |
| OR1A1 | - | 0.055 | - | 0.305 |
| OR1C1 | - | 0.175 | - | 0.054 |
| OR1D2 | - | 0.437 | - | 0.361 |
| OR1G1 | - | 0.206 | - | 0.122 |
| OR2A25 | - | 0.272 | Inhib | 0.010 |
| OR2AG1 | - | 0.143 | - | 0.201 |
| OR2AG2 | Activ | 0.012 | - | 0.332 |
| OR2AT4 | - | 0.292 | - | 0.173 |
| OR2B6 | - | 0.489 | - | 0.484 |
| OR2B11 | - | 0.107 | - | 0.442 |
| OR2C11 | Activ | 0.007 | - | 0.284 |
| OR2D2 | - | 0.081 | Inhib | 0.028 |
| OR2D3 | Activ | 0.006 | Inhib | 0.046 |
| OR2G2 | Activ | 0.032 | Inhib | 0.045 |
| OR2J2 | Activ | 0.035 | Inhib | 0.047 |
| OR2J3 | Inhib | 0.013 | Inhib | 0.000 |
| OR2T1 | Inhib | 0.018 | Inhib | 0.027 |
| OR2T4 | - | 0.368 | Inhib | 0.005 |
| OR2T6 | - | 0.401 | - | 0.157 |
| OR2T7 | - | 0.073 | - | 0.057 |
| OR2T8 | Inhib | 0.015 | Inhib | 0.038 |
| OR2T11 | - | 0.130 | - | 0.180 |
| OR2T29 | - | 0.495 | - | 0.479 |
| OR2W1 | Inhib | 0.041 | - | 0.104 |
| OR5A2 | - | 0.290 | Inhib | 0.006 |
| OR4C15 | - | 0.147 | Inhib | 0.000 |
| OR4E1 | Activ | 0.022 | Inhib | 0.006 |
| OR4E2 | - | 0.328 | Inhib | 0.009 |
| OR4P4 | - | 0.130 | Inhib | 0.033 |
| OR4Q3 | - | 0.090 | - | 0.127 |
| OR5A1 | Activ | 0.050 | - | 0.059 |
| OR5AN1 | - | 0.068 | - | 0.102 |
| OR5B3 | - | 0.450 | Inhib | 0.002 |
| OR5I1 | - | 0.058 | - | 0.105 |
| OR5K1 | - | 0.053 | - | 0.081 |
| OR5K3 | - | 0.279 | Inhib | 0.013 |
| OR5L1 | Activ | 0.006 | - | 0.090 |
| OR5P3 | Activ | 0.001 | - | 0.062 |
| OR6A2 | Activ | 0.023 | - | 0.068 |
| OR6P1 | Activ | 0.026 | Inhib | 0.023 |
| OR7C1 | Activ | 0.035 | - | 0.378 |
| OR7D4 | - | 0.056 | - | 0.351 |
| OR8B3 | Activ | 0.024 | Inhib | 0.002 |
| OR8D1 | - | 0.078 | Inhib | 0.020 |
| OR10A2 | - | 0.120 | Inhib | 0.038 |
| OR10A4 | Inhib | 0.031 | - | 0.059 |
| OR10A5 | - | 0.477 | - | 0.287 |
| OR10A6 | - | 0.368 | Inhib | 0.028 |
| OR10G3 | Activ | 0.022 | Inhib | 0.045 |
| OR10G4 | Activ | 0.035 | - | 0.137 |
| OR10G7 | Activ | 0.033 | Inhib | 0.010 |
| OR10H4 | Activ | 0.021 | Inhib | 0.002 |
| OR10J5 | Activ | 0.031 | - | 0.159 |
| OR11G2 | - | 0.057 | Inhib | 0.013 |
| OR11H6 | Activ | 0.015 | Inhib | 0.003 |
| OR13A1 | Activ | 0.008 | Inhib | 0.031 |
| OR51D2 | - | 0.376 | Inhib | 0.042 |
| OR51E1 | Inhib | 0.005 | Inhib | 0.028 |
| OR51E2 | Inhib | 0.007 | Inhib | 0.012 |
| OR51L1 | - | 0.389 | Inhib | 0.020 |
| OR52A1 | - | 0.062 | - | 0.063 |
| OR56A4 | Activ | 0.008 | - | 0.358 |

**S1 Table. Screening for human olfactory receptor Activation by tears in vitro.**

The table summarizes the screening after olfactory receptor activation in vitro, of 62 human ORs, induced by tears and trickled saline. The results are depicted in Figure 1. P values of one-tail paired t-test comparison between solvent (CD293 medium) to 3.6% (v/v) tears or saline. Activ= considered as activated receptor, Inhib = considered as inhibited receptor. The corresponding p-values depicted in the table.
