# Supplementary material for: A chemical signal in human female tears lowers aggression in males
Source: PLoS Biol. 2023 Dec 21;21(12):e3002442. doi: 10.1371/journal.pbio.3002442 (PMC10734982; doi:10.1371/journal.pbio.3002442)
Supplement: S2 Table — Repeated measures ANOVA for OR dose–response activation assay. F statistic and P value matched for the compound factor (saline vs. tears). (DOCX) [file pbio.3002442.s015.docx]

| OR subtype | Number of repetitions | F statistic | P-value |
| --- | --- | --- | --- |
| OR11H6 | 3 | F(1,28) = 40.99 | P < 0.0001 |
| OR2AG2 | 3 | F(1,28) = 22.65 | P < 0.0001 |
| OR5A1 | 3 | F(1,28) = 5.827 | P = 0.02 |
| OR2J2 | 3 | F(1,28) = 17.2 | P = 0.0003 |

**S2 Table. OR dose-response statistics**

Repeated measures ANOVA for OR dose-response activation assay. F statistic and P-value matched for the compound factor (saline vs. tears).
