# Supplementary material for: A chemical signal in human female tears lowers aggression in males
Source: PLoS Biol. 2023 Dec 21;21(12):e3002442. doi: 10.1371/journal.pbio.3002442 (PMC10734982; doi:10.1371/journal.pbio.3002442)
Supplement: S3 Table — Coordinates and Z-statistics for all significant activation (P < 0.001, corrected for multiple comparisons P < 0.05) for the contrast Provocation > inactive time. (DOCX) [file pbio.3002442.s016.docx]

Provocation vs. inactive time

| Area | Voxels | P | Z | Peak activation coordinates | | |
| --- | --- | --- | --- | --- | --- | --- |
|  |  |  |  | **x** | **y** | **z** |
| Right Occipital Lobe, including:  Lateral Occipital Cortex  Fusiform Gyrus | 64969 | 4.07 e^-32^ | 6.41 | 28 | -68 | 40 |
| Left Lateral Occipital Cortex | 44139 | 1.01 e^-24^ | 6.23 | -24 | -77 | 23 |
| Right Frontal area, including:  Middle Frontal Gyrus  Prefrontal Cortex  Precentral Gyrus  Inferior Frontal Gyrus | 10899 | 1.81 e^-09^ | 5.41 | 49 | 7 | 43 |

**S3 Table. ﻿Brain areas activated by provocation events compared to baseline**

﻿Coordinates and Z-statistics for all significant activation (P < 0.001, corrected for multiple comparisons P < 0.05) for the contrast Provocation > Baseline.
