# Supplementary material for: A chemical signal in human female tears lowers aggression in males
Source: PLoS Biol. 2023 Dec 21;21(12):e3002442. doi: 10.1371/journal.pbio.3002442 (PMC10734982; doi:10.1371/journal.pbio.3002442)
Supplement: S4 Table — Coordinates and max intensity Z-statistics for all significant activation (P < 0.005, corrected for multiple comparisons P < 0.05) for the contrast Provocation > inactive time and saline > tears. (DOCX) [file pbio.3002442.s017.docx]

Provocation, Saline > Tears

| Area | voxels | P | Z | Peak activation coordinates | | |
| --- | --- | --- | --- | --- | --- | --- |
|  |  |  |  | **x** | **y** | **z** |
| Occipital area, including:  R-Lingual gyrus  L- Fusiform gyrus  L-Lateral occipital cortex, inferior division | 31176 | 1.77 e^-15^ | 4.44 | 21 | -57 | -15 |
| R-Prefrontal cortex | 10301 | 7.15 e^-07^ | 3.89 | 48 | 44 | 11 |
| R-Superior parietal lobe | 6857 | 5.41 e^-05^ | 4.25 | 33 | -50 | 36 |
| L- Prefrontal cortex | 5306 | 0.000476 | 3.6 | -30 | 41 | 21 |
| L-Supramarginal gyrus | 5159 | 0.00059 | 3.75 | -55 | -37 | 48 |
| Precentral gyrus | 4732 | 0.00112 | 3.99 | 8 | -5 | 74 |
| R-Cerebellum, viib | 4255 | 0.00234 | 3.8 | 20 | -72 | -58 |
| L-Heschl’s gyrus | 3134 | 0.0149 | 3.87 | -50 | -14 | 8 |
| L-Anterior insular cortex | 2729 | 0.0305 | 3.76 | -27 | 23 | 4 |
| L-Cerebellum, vii | 2505 | 0.0459 | 3.97 | -41 | -56 | -60 |

**S4 Table. ﻿Brain areas activated by provocation events compared to baseline and saline compared to tears**

﻿Coordinates and max intensity Z-statistics for all significant activation (P < 0.005, corrected for multiple comparisons P < 0.05) for the contrast Provocation > Baseline and saline > tears.
