# Supplementary material for: A chemical signal in human female tears lowers aggression in males
Source: PLoS Biol. 2023 Dec 21;21(12):e3002442. doi: 10.1371/journal.pbio.3002442 (PMC10734982; doi:10.1371/journal.pbio.3002442)
Supplement: S5 Table — The mean time from the beginning of provocation event to the end of the monetary response in which it was nested. This time was specified as provocation event in the fMRI GLM analysis for each subject. Time in seconds of both sessions (day 1 and day 2) are depicted. (DOCX) [file pbio.3002442.s018.docx]

| Subject ID | Mean time from provocation  onset to monetary offset (sec) | |
| --- | --- | --- |
|  | Session 1 | Session 2 |
| AO822 | 5.9 | 5.9 |
| BA093 | 1.8 | 2.0 |
| BMA339 | 5.9 | 3.9 |
| BO463 | 1.6 | 1.8 |
| BTD643 | 2.7 | 2.3 |
| BYO847 | 5.3 | 5.8 |
| CR942 | 5.9 | 5.7 |
| DG778 | 2.2 | 2.5 |
| DY313 | 5.8 | 5.9 |
| GB409 | 6.1 | 5.8 |
| GTI792 | 6.0 | 5.8 |
| HA134 | 5.8 | 5.8 |
| HA572 | 6.0 | 5.6 |
| HD516 | 5.3 | 5.2 |
| IG129 | 1.7 | 1.6 |
| KH601 | 6.0 | 5.7 |
| KS959 | 5.8 | 5.9 |
| NY249 | 5.0 | 5.7 |
| PA005 | 5.9 | 5.8 |
| PR454 | 6.0 | 5.6 |
| RO100 | 3.3 | 5.2 |
| SS952 | 6.0 | 5.8 |
| TYR151 | 2.4 | 3.4 |
| ZD734 | 5.7 | 5.8 |

**S5 Table. Time from provocation onset to monetary offset**

The mean time from the beginning of provocation event to the end of the monetary response in which it was nested. This time was specified as provocation event in the fMRI GLM analysis for each subject. Time in seconds of both sessions (day 1 and day 2) are depicted.
