# Supplementary material for: A chemical signal in human female tears lowers aggression in males
Source: PLoS Biol. 2023 Dec 21;21(12):e3002442. doi: 10.1371/journal.pbio.3002442 (PMC10734982; doi:10.1371/journal.pbio.3002442)
Supplement: Attachment — Submitted filename: AgronRefereeResponse.pdf [file pbio.3002442.s019.pdf]

To Referee 1
We thank you for a very careful and very constructive review. We concurred with all comments made, and modified the manuscript accordingly. The details of our revision follow:

Reviewer #1, Keith M Kendrick (note, Reviewer 1 has signed this review):

This is an intriguing follow up study by this group reporting that exposure to the emotional tears of women reduces aggressive behavior in men during performance of an economic game. Their behavioral findings are complimented by additional ones showing that some olfactory receptors can respond dose dependently to the womens' tears in vitro and that reduced activity occurred in some olfactory/aggression processing brain regions when men were exposed to the tears in an MRI scanner. Overall, the study is interesting and thorough as well as technically challenging and the paper is well written.

We thank the Referee for the kind words

I do have some comments however which I think the authors should address:

We numbered the questions, and address constructively:

1. I think that to state the tears are odorless is technically incorrect since they appear to be able to influence the olfactory system and the whole claim is that the reduction in aggression is due to the olfactory cues in the tears. What does appear to be true is that the odors from the tears themselves cannot be consciously discriminated by human males. This is perhaps somewhat akin to subliminal processing of visual cues which can influence behavior but without being consciously perceived.

We appreciate this incite, concur, and have modified the text accordingly throughout, highlighting the distinction between perception and conscious perception. More specifically, we deleted 7 instances of the word “odorless”, and in the remaining use of the term, modified to “perceptually odorless”.

2. I find myself struggling a little to find a context in which the odor of tears might influence a man's behavior given that in humans both visual and auditory cues for crying are far easier for us to perceive.

Note in passing: We could not help but laugh between us in writing the answer here, because there is a famous Israeli song entitled and stating that “men cry at night and their voice cannot be heard”.... (https://www.youtube.com/watch?v=s2atQAQF-Yk) But this is besides the point:-)

It is much simpler to conceptualize this type of signaling in nocturnal animal species where individuals live in close proximity to one another, and of course also have a functional vomeronasal system. I think it would be useful if the authors at least speculate in what contexts the odor of women's tears might be relevant. At this point we also don't know if the sight of tears or the sounds of crying might produce similar aggression reducing effects.

We appreciate these comments, and can speculate in their view, but stress that this is speculation alone: Crying can occur is very close contact. Indeed, across cultures, it is common to "kiss crying cheeks". In other words, it is in fact common to even taste tears, let alone be close enough to sniff them. Moreover, although here we tested tears obtained from women donors, we speculate the response would be the same to any tears, and particularly infant tears. Here the ecological relevance is much greater. Infants have no language they might use to curb aggression towards them, and therefore must rely on non-verbal messages. The sound of crying babies can actually even increase aggression [1], therefore, a chemosignal that can lower aggression is highly valuable for them. Moreover, interactions with infants are also from close distance. As to the impact of seeing tears, such sight indeed impacts emotions, particularly empathy [2], but an impact on aggression has not been directly tested. We of course want to limit speculation in the manuscript, yet we now moderately eco these considerations in the discussion.

3. The authors measured state anxiety and trait aggression using STAI and Buss-Perry questionnaires after the experiment. It would be useful to know to what extent these measures correlate with aggression scores on the PSAP. Stimuli which evoke aggression often do so mainly in individuals who have high trait aggression so it would be interesting to know if the effects of tears in reducing aggression were greatest in individuals with higher trait aggression.

This is an insightful possibility. To address it, we now calculated the correlation between the APR score (tears - saline) and the TA and AGQ scores. In experiment 3 (MRI), the correlation between AGQ anger component and APR difference was $r = -0.51$, $p = 0.011$. However, after correcting the p for multiple comparisons, it was only close to significant ($p = 0.055$). Thus, we did not include this result in the main manuscript. Also, our findings revealed no significant correlation between questionnaire scores and behavior in the PSAP game. We now added Supplementary Figure 8 that depicts the results of the correlation between the delta in APR scores (tears - saline) to the AGQ and TA scores for all participants in Experiment 1 and Experiment 3 together.

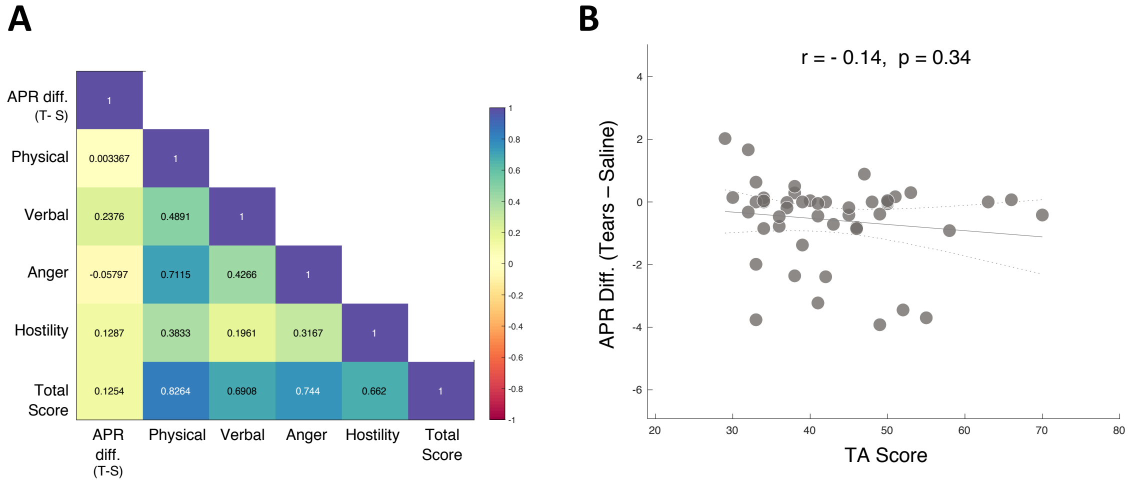

Supplementary Figure 8. Trait aggression and state anxiety, as measured by AGQ and STAI, respectively, did not correlate with the effect of tears on aggressive behavior. **A.** Correlation matrix of AGQ; total score and the different factors with the APR difference between stimuli (Tears – Saline), n= 49. The color bar represents the Spearman's rank correlation coefficient (r), also depicted. All p-values > 0.5 (corrected for multiple comparisons). **B.** Correlation between STAI score and the APR difference between stimuli (Tears – Saline). Each dot represents a participant, n=47 (two participants did not completed this questionnaire). The continuous line represents the fit. The dashed line marks the confidence bounds. Spearman's rank correlation coefficient and p-value are depicted.

4. The authors excluded one subject as a statistical outlier based on the standard definition of an outlier using IQ ranges, however they also used non-parametric statistics due to non-normal distribution which generally makes data analysis insensitive to outliers, at least in terms of type 1 errors. Does inclusion of this subject prevent achievement of a significant p value?

If we include the outlier, the effect remains. More specifically: with the outlier, the mean APR following trickled saline was 1.79±1.78, APR following tears was 1.11±1.28 (Shapiro-Wilk, $W = 0.831$, $p < 0.001$, implying a non-normal distribution dictating a nonparametric test: Wilcoxon signed rank $Z = 66$, $p = 0.05$, Cohen's $d = 0.48$, with no effect of order: Wilcoxon signed rank $Z = 115$, $p = 0.72$, Cohen's $d = 0.091$. If we nevertheless use the parametric approach, the effect remains the same: $t(25) = -2.5$, $p = 0.019$, Cohen's $d = 0.491$, with no effect of order: $t(25) = -0.441$, $p = 0.663$). We added this information to the revised text.

5. Minor point: On page 15, line 9 "immerged" should be "emerged"

Thanks, corrected

To Referee 2
We thank you for a very careful and very constructive review. We concurred with all comments made, and modified the manuscript accordingly. The details of our revision follow:

Reviewer #2:
General comments
The authors tested the influence of human tears on behavioral aggression, olfactory receptors, and fMRI activity in aggression networks. The approach is innovative and yields intriguing results in an area that is so far understudied in humans.

We thank the Referee for the kind words

However, there are some significant issues that the authors should address to increase confidence in the results. The biggest sticking point is related to experiment 3, where there are concerns about the way the behavioral results are reported, and a significant imbalance across conditions following exclusions. Other concerns are mainly related to the narrative structure of the article, and errors/inconsistencies/gaps in reporting, which are more easily addressed.

We here address constructively:

Specific comments

Major comments
1. * The introduction focuses on biological mechanisms of chemical signaling (e.g., the role of ESP1), which is interesting and somewhat relevant. However, given the nature of the study, shifting the introduction's focus toward behavioral outcomes, especially in humans, would provide helpful context. For instance, the authors could briefly report behavioral and fMRI findings from Gelstein et al., 2011 (from the same group), Mishor et al., 2021 (also from the same group), and Oh et al., 2012, and discuss the known fMRI correlates of aggression.

We have modified the introduction to now also discuss the human behavioral and brain response to tears, and to manipulations of aggression. Moreover, we discuss Mishor et al and related materials in the discussion.

2. * Behavioral findings from experiment 3 should be reported in a similar manner to those from experiment 1 (i.e., by comparing APR values across conditions for the entire group, and plotting the data in figures similar to those in Figure 1 [D-F]).

We concur, and have changed the relevant figures accordingly.

3. Although it's plausible that order effects could be accentuated in the MRI scanning context, properly counterbalancing the conditions should account for this issue. However, based on data file 4, it seems that the groups were no longer well-balanced following exclusions, with 11 participants in the saline-first group, and 15 participants in the tears-first group. If order plays

a significant role in modulating aggression as the authors suggest, this is a significant problem, since differences in fMRI correlates of aggression could be driven by the order of conditions, rather than the chemical stimulus applied. Ideally, the authors would recruit more participants to balance the groups, or at least account for the imbalance somehow in their models.

This is a keen observation with which we concur. To address this potential problem and balance the groups, we conducted the following analyses:

We have 9 fMRI participants who had saline-first. Thus, a balanced group from this perspective is reduced to 18 participants, which is borderline in power. To overcome this, we conducted two analyses. First, using a bootstrap approach, we randomly selected balanced groups of 18 participants 10,000 times, and each time conducted the analysis to create a distribution. Had we done this on the entire brain it would have taken a few months to run, therefore we limited this analysis to the two ROIs we reported in the study, namely the AIC and PFC. In a second analysis we made a one-time selection of a balanced group of 18, and conducted full brain analysis. Here we had to first decide which tears-first participants we ignore, and did this by removing the low AGQ scoring individuals to maintain two AGQ balanced groups. Both of these tests confirmed our previous findings. The bootstrap analysis revealed an average $t(17) = 2.414$, p-value = 0.037 for the left AIC, and an average $t(17) = 3.815$, p-value = 0.0015 for the PFC. New Supplementary Figure 6A-B provides additional details. The whole brain analysis of the counterbalanced group (n=18) is available in new Supplementary Figure 6C. Furthermore, the correlation between behavior and brain response in the ROIs remained consistent even in the smaller group, as shown in Supplementary Figure 6D-E.

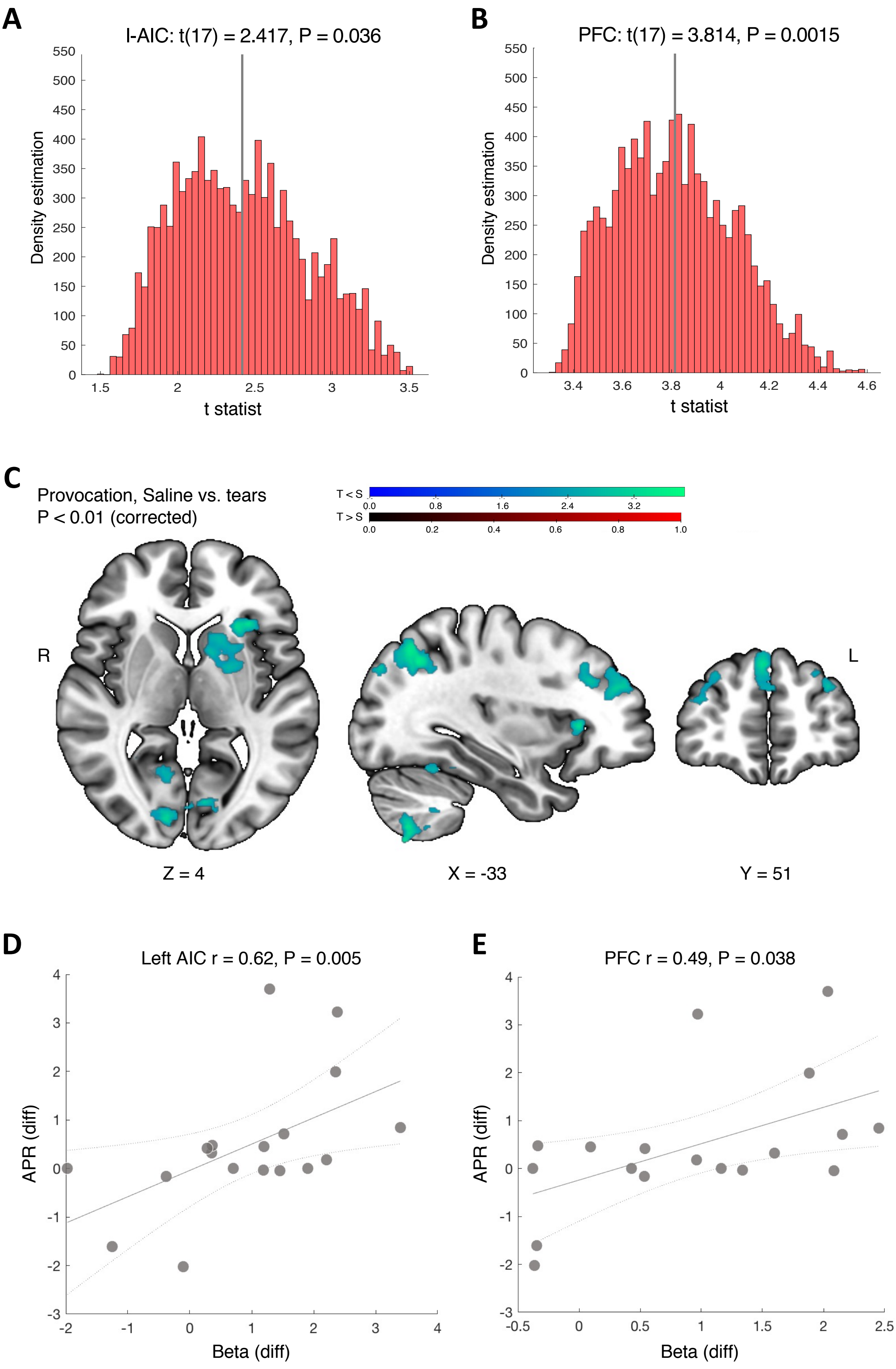

Supplementary Figure 6. Tears' effect remains after counterbalancing for stimuli order.

In the bootstrap analysis, participants were randomly selected 10,000 times to counterbalance for stimuli order. Beta values for **A.** left AIC and **B.** PFC were then compared for each stimulus. The histograms represent the distribution of the t statistic of all repetitions for each ROI. The grey horizontal line represents the mean statist. The mean t statists and the p values are depicted. **C.** Statistical map of the GLM ANOVA Provocation with an added level of saline vs. tears (tears < saline in blue; tears > saline in red), counterbalanced for order, n=18. z threshold > 2.31, cluster corrected to p = 0.05. Color bars represent z-values. Correlation between differences in behavioral APR scores (saline -tears) and differences in beta values (saline- tears) of **D.** left AIC and **E.** PFC. Each dot represents a participant. The continuous line represents the fit. The dashed line marks the confidence bounds. Spearman's rank correlation coefficient and p-values are depicted.

4. * Although results from this study could be related to COVID-19, this is very speculative. It's not clear that anosmia in COVID patients results in a parallel deficit in "odorless" chemical signaling, and there are a host of other (probably more salient) reasons why aggression might manifest in individuals with a complex illness. Drawing conclusions about men in particular seems inappropriate, given that there's no gender comparison in this study. Perhaps the authors could bring up the COVID-19 link, but in a more measured way, and without emphasizing it.

Agreed – removed completely

Minor comments

5. * Were participants asked whether they believed their PSAP opponent was human? If not, this should not be assumed in the results section.

To ensure the integrity of the experiment's paradigm, we did not ask participants directly if they believed they were playing against a real opponent before finishing collecting the data. Instead, we collected information indirectly by asking participants about their thoughts and feelings towards their opponent, both verbally and through a questionnaire at the end of the experiment. We only excluded one participant who expressed doubt about his opponent's human identity, as social interaction is a crucial element of our study on social chemosignals. We now added further clarification in the methods section (see Questionnaires).

6. * It's stated that participants did not know they were sniffing tears during the sniff-jar procedure. What were the participants told, if anything, about the stimulus?

Our IRB document that participants sign, and are supposed to read before participation (although few if any read it carefully) noted that "as a participant you may receive assorted odors, including body odors, and at various intensities, above and below threshold". In other words, they generally know (and agree) that sniffing body odors is a "possibility" they may encounter in the study, but this is not up front in their attention.

7. * Could the authors include bar plots to illustrate the mean pleasantness, intensity, and familiarity for tears vs. saline in Figure 1? There's also an inconsistency on the plot (which

references experiments 1 and 2) and the legend and supplementary data file (which reference experiments 2 and 3) - from reading the manuscript, I believe that all of these should reference experiments 1 and 3. Most importantly though, the idea that the tears are "odorless" is not supported by the data, since participants rate the tear stimuli as moderately intense. It would be more accurate to say that previous studies (e.g., Gelstein et al., 2011) have demonstrated that tear and saline stimuli cannot be discriminated based on odor, which implies that the chemical signal contained in tears is odorless. The data collected here support this idea (since there aren't meaningful differences in ratings across stimuli), but it is not tested directly.

Thanks for drawing our attention, we fixed this typo and added a bar plot in Supplementary Figure 2A-C. We would like to stress that the tears are perceptually odorless. The “moderate intensity” ratings applied by participants are merely because human raters rate a non-stimulus as moderate in such tests. This is clearly seen in that saline was also rated in the same way, and saline is clearly odorless. It has no VOC in it. We could have presented the data as “difference from saline”, and then tear intensity would be at zero, yet we opt to show the rating data as is. Indeed, both scatter and bar plots show that tear ratings did not differ from those of trickled saline in all tested parameters, including intensity. Moreover, both stimuli (trickled saline and tears) odor descriptor ratings did not differ from blank (not trickled) saline solution (we added this information to the text and to Supplementary Figure 2D-F).

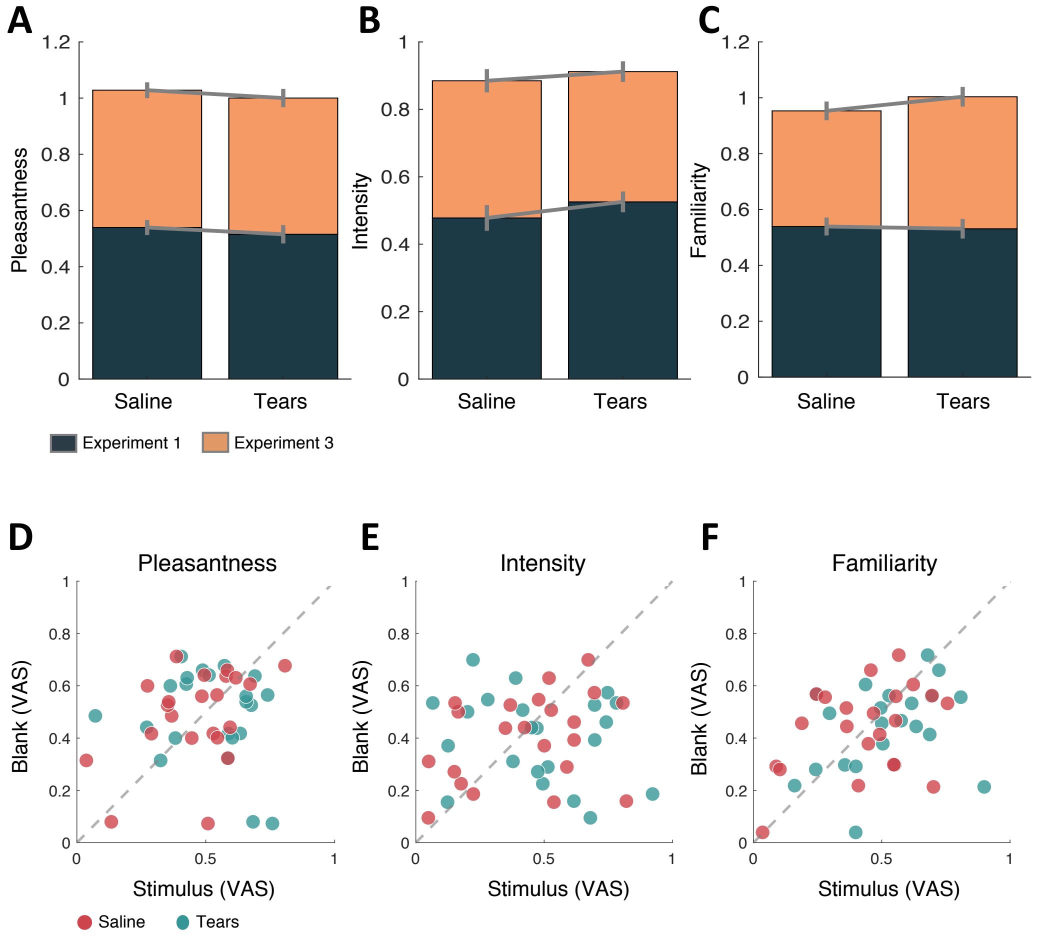

Supplementary Figure 2. Tears and trickled saline were not perceptually discriminable from a blank saline solution.
The bar plot depicts the mean ratings of A. pleasantness B. intensity, and C. familiarity of tears vs. trickled saline. Whiskers represent the SE. (the data of Experiment 1 in dark and Experiment 3 in light color). The scatter plots depict of the normalized VAS ratings of saline solution (blank) and stimulus (tears in blue and trickled saline in red) for D. Pleasantness, E. Intensity, and F. Familiarity. Each dot represents the average of 6 blank sniffs and 10 stimulus sniffs (normalized to min max values) of each participant. The data in D-F are presented along a unit slope line (X=Y), such that if points accumulate above the line, this implies higher values after tears, if points accumulate below the line, this implies higher values after saline, and if points are distributed around the line, this implies no difference.

8. * The APR is a ratio, but several participants have a score of 0 for one or both conditions. What does a 0 value mean in this case? And do the numbers following the mean APR (e.g., 1.67+/-1.7 APR) represent the standard error?

A score of zero means no aggressive response (0 revenge response divided by the number of provocations). The numbers following the mean APR are standard deviation (SD) and not standard error, we added this clarification to the text.

9. * It does not seem appropriate to include OR1A1 in the olfactory receptor analysis, given that it does not meet the established criteria, and there are other ORs that are similarly close to the cutoff, but are not included (e.g., OR5K1). If the authors feel that it's important to include this receptor, then they should explain why in the main text (beyond stating general interest), and this should be framed as a separate exploratory analysis (after the main analysis). Could the authors also provide justification for moving forward with receptors that are activated, but not inhibited, by the stimuli (given that inhibition could also occur in a dose-dependent manner)?

We concur that retaining OR1A1 in the manuscript does not meet strict statistical inclusion criteria, and therefore we removed it. It was indeed there because we have an extended interest in it from a different study, but this is indeed irrelevant. Finally, two ORs, 2W1 and 10A4, were inhibited by tears and not by saline in our screening assay. However, this inhibition was not confirmed by the followed dose response assay.

10. * The authors state that the order of conditions was counterbalanced for experiment 1, and time of day was held constant. Can the authors clarify (in the main text) whether this was the case for experiment 3 as well?

Yes, this was the case in 3 as well, but the balance in analysis was violated following exclusions. We have now addressed this violation in Supplementary Figure 6, and find that it did not introduce or eliminate any effects.

11. * The authors state: "The contrast of provocation vs. baseline regardless of condition revealed a typical salience network activation, which included typical provocation-associated regions." Framing the control condition as a "baseline" seems counterintuitive, since it implies that there's no contrast. Can the authors use another label for the control condition in the main text and associated figures to avoid confusion?

The "baseline" we refer to in this sentence is not the saline condition, it is the condition in the PSAP that is "not Provocation". We have modified the text to better clarify this.

12. * The authors should add p-value thresholds to MRI figure legends and cluster sizes to supplementary MRI tables for more complete information.

We added this information to the revised manuscript.

13. * The authors should make it clear whether the difference metrics represented in Figure 3 B-C are subtracting the saline values from tears values, or vice versa (so the direction of the correlation is well-defined).

Thanks for the comment. This is now specified in the revised text.

14. * Was the olfactory cluster the only cluster to emerge in the functional connectivity analysis? If so, this should be clarified. If not, the authors should provide a supplementary table that lists the brain regions observed (as they did for prior analyses).

Yes. This is now clarified in the text. "We observed that tears significantly impacted functional connectivity only for the left AIC, which under tears significantly increased connectivity specifically with the right temporal pole (right TP) extending into the right amygdala and piriform cortex"

15. * Although results suggest that neural responses are decreased in the tear condition, and functional connectivity with an olfactory-adjacent region is increased, the authors should refrain from implying that one result causes the other (e.g., "We also find that this signal increases functional connectivity between the brain substrates of olfaction and aggression, reducing overall levels of activity in the aggression brain network.")

We did not intend to claim causality between these independent findings within our data. To stress this, we now added "we acknowledge no causal link between these findings within our data".

16. * Why were participants exposed to "blanks" (saline?) for 3 sniffs prior to the 10 main sniffs in experiment 1, and then not in experiment 2?

The added 3 "blank" sniffs are of saline that was not trickled on cheeks. This allows us to test for any perceptual differences between trickled saline and saline alone. We only need to do this

once (there was no difference). Note, the three added sniffs were there for both conditions, so this does not introduce any experimental difference or imbalance. See Supplementary Figure 2D-F.

17. * For the various questionnaires the participants completed, could the authors report the rationale for administering these and/or the main results? For instance, participants completed an autism quotient questionnaire - why?

In a previous study we observed significantly altered social chemosignaling in ASD [3], therefore, autism quotient is an important measure in such studies. Our participants are typically graduate students at the Weizmann Institute. We will share here with the referee that for every cohort of ~30 we run, we typically encounter at least one participant who would meet the diagnostic criteria for ASD.

Participants also completed anxiety and aggression questionnaires, and rated how much they would want to meet their opponent. Reporting the difference (or lack of) across conditions could be informative.

We agree that this is potentially an interesting analysis, and now ran it. We find that participants did not rate their opponent differently between conditions (Supplementary Figure 7), and there was no correlation between the participants' APR scores and their innate aggressive traits or anxiety state (new Supplementary Figure 8).

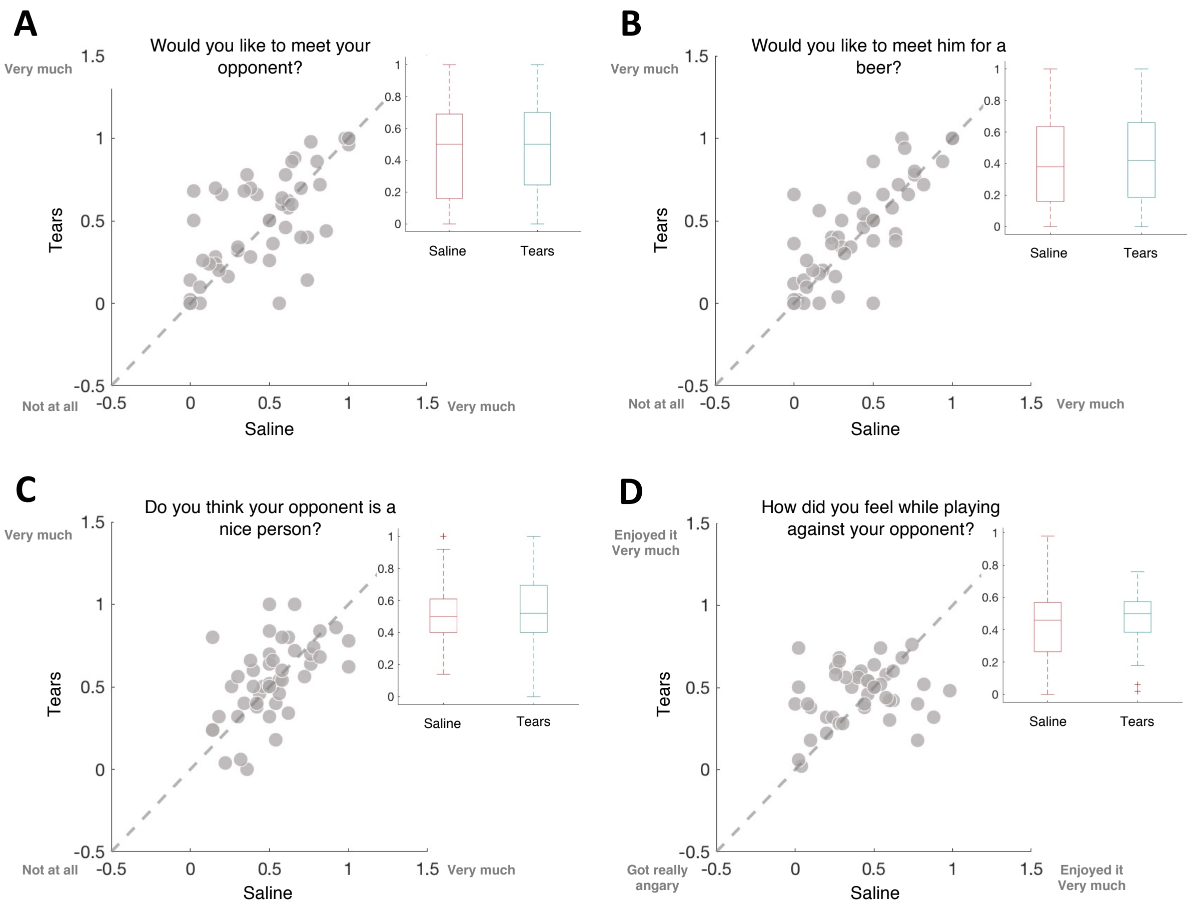

Supplementary Figure 7. Participants' social attitude towards their opponent was not affected by tears.

Participants completed a questionnaire that included social questions regarding their opponent at the end of each experimental day (i.e., once after sniffing tears and once after sniffing saline). Participants answered the following questions using a VAS ranging from "very much" to "not at all" : A. Would you like to meet your opponent? (Shapiro-Wilk, $W = 0.94$, $p < 0.03$, implying a non-normal distribution dictating a nonparametric test: Wilcoxon signed rank $Z = 330$, $p = 0.195$, corrected $p = 0.78$). B. Would you like to meet him for beer? (Shapiro-Wilk, $W = 0.92$, $p = 0.003$, implying a non-normal distribution dictating a nonparametric test: Wilcoxon signed rank $Z = 293$, $p = 0.077$, corrected $p = 0.3$). C. Do you think your opponent is a nice person? ($t(50) = -0.816$, $p = 0.42$, corrected $p > 0.99$). D. How did you feel while playing against your opponent? Participants answered this question using a VAS ranging from "enjoyed it very much" to "Got really angary", (Shapiro-Wilk, $W = 0.944$, $p < 0.02$, implying a non-normal distribution dictating a nonparametric test: Wilcoxon signed rank $Z = 284$, $p = 0.14$, corrected $p = 0.56$). Participants did not show a difference in their social attitude towards their opponent across stimuli.

18. * Participants also donated saliva. Why did they do this? Was the testosterone content analyzed?

Originally, we planned to measure testosterone levels in salivary samples before and after the PSAP game. The testosterone assay must be conducted for all data simultaneously at the end of the study, using the same plate, and not subject by subject. Here, in the middle of the study, we were hit by COVID, and could not analyze saliva. We ended up with samples in the freezer for a very very long time (years), and judged that it would be wrong to run assays on this material. Its still in the freezer… We now detail this in the manuscript.

19. * Given that participants sniffed "blanks" prior to the 10 main sniffs in experiment 1, it does not seem necessary to remove the first sniff rating from the analysis. Could the authors report whether the results change if the first rating is included? There are also contradictions here, since the ratings were normalized to min-max values within all twenty odor exposures, and Figure 1 references the average of ten sniffs by a given participant.

We re-analyzed the data keeping the first sniff after the blank. Again, we observed no perceptual differences between tears and trickled saline, which did not significantly differ in perceived intensity, pleasantness, and familiarity ($F(1,20) = 1.923$, $p = 0.18$) (Figure 2A-C) (Data File 1). As for the normalization process, we applied min-max normalization to the data from both days to identify any differences between the two stimuli. This involved normalizing 20 sniffs, consisting of 10 tears and 10 saline samples. Once the normalization was complete, we used a scatter plot to compare and contrast the mean values of tears and saline. We changed the text in the manuscript for clarification.

20. * For participants who were excluded for being outliers, was this based on their APR scores? Could the authors report these scores (at least in the methods and data files, and ideally in the main figure legends) for completeness? It's also unclear why a participant would be excluded for reporting that he did not believe he played against a real opponent. Again, was this probed specifically? Or based on an offhand comment? If it's the latter, it's possible that other participants felt similarly, even though they did not express it.

Yes, outliers were excluded based on their APR score. The scores of the outliers are now added to the data files (Data File 2 and Data File 4). Participants were asked to provide their overall impression of the game and write about their opponent using their own words in a post-experiment questionnaire without explicitly asking about the opponent's identity. They were also asked about it verbally by the experimenter. Though it is possible that other participants felt the same, only one indicated doubts about his opponent's human identity. Since we tested tears in the context of social chemosignals, believing in playing against a real person opponent is relevant to the context, Thus, we think it is more adequate to exclude him from the analysis [4-6]

21. * Could the authors clarify the timeline for the PASP in experiments 1 and 3? In the main text it's implied that this took 2 hours, but the authors mention a single MRI run (per day) in the methods.

For both experiments, each session lasts approximately two hours every day. In experiment 3, around 45 minutes of the session were spent in the scanner. We made it clearer in the text.

22. * Could the authors report whether there are statistically significant differences in mean time from provocation onset to monetary offset for day 1 vs. day 2, and for tears vs. saline?

There are no significant differences in the average time from provocation onset to monetary offset between days or between stimuli. This is now depicted in Supplementary Figure 10.

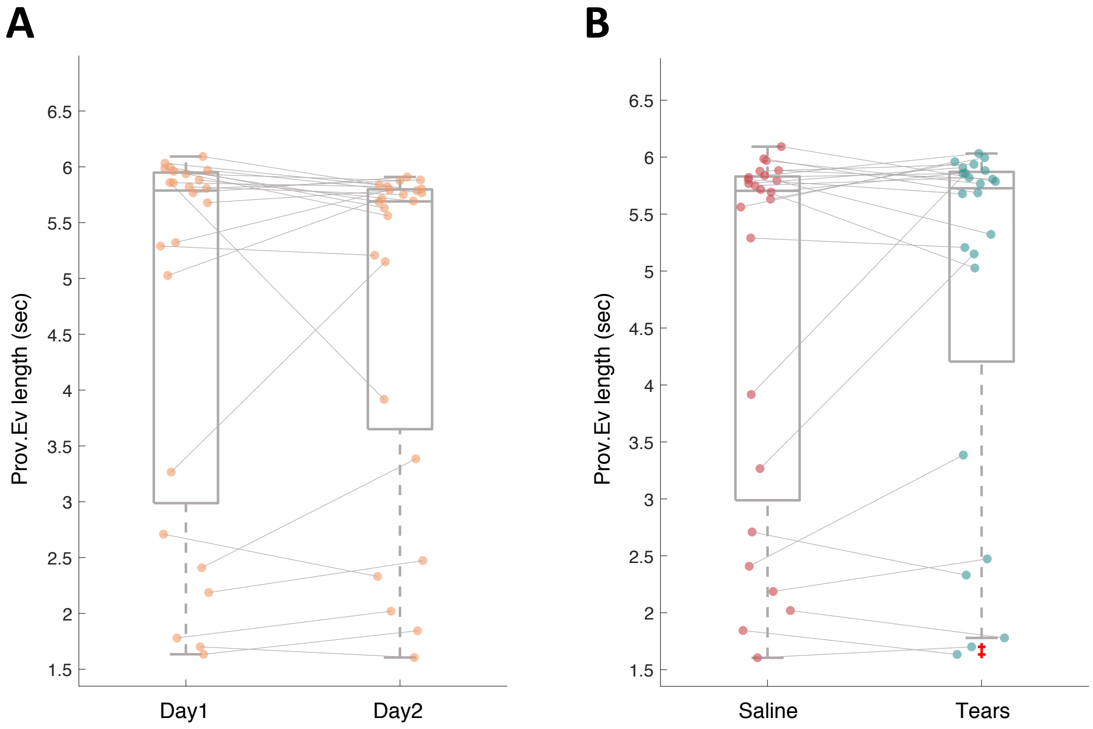

Supplementary Figure 10. **Provocation event length was constant for each participant throughout the different days and stimuli.**
A. No difference in provocation event length between days (Shapiro-Wilk, W = 0.817, $p < 0.001$, implying a non-normal distribution dictating a nonparametric test: Wilcoxon signed rank Z = 156, p = 0.86, effect size (r_{rb}) = 0.16). **B.** No difference in provocation event length between stimuli, saline in red and tears in blue (Shapiro-Wilk, W = 0.78, $p < 0.001$, implying a non-normal distribution dictating a nonparametric test: Wilcoxon signed rank Z = 142, p = 0.95, effect size (r_{rb}) = 0.048). The rectangle reflects the upper and the lower interquartile (25th to the 75th percentiles) and the whiskers are minimum and maximum non outlier (no more than 1.5 * IQR of the upper and lower hinges). Outlying points are plotted individually. The line inside the box is the sample median. Each point is a participant, and the line connects the repeated measure.

23.* The methods reference regressing out volumes according to frame-wise displacement of greater than 0.9. Is this 0.9 mm?

Yes, we added the units to the text.

24.* The authors state that they conducted whole-brain PPI analyses using three ROIs as seeds in the methods, but then only name two of these ROIs (left AIC and PFC).

This was a typo, as evident even within the original sentence: "We conducted a whole brain PPI analysis using three ROIs that emerged from the group analysis contrast of saline vs. tears as seeds to explore functional connectivity with these regions during provocation (left AIC and PFC)". Left AIC and PFC are indeed **two** regions, and the sentence should have been: "We conducted a whole brain PPI analysis using two ROIs that emerged from the group analysis contrast of saline vs. tears as seeds to explore functional connectivity with these regions during provocation (left AIC and PFC)". We corrected.

To Referee 3
We thank you for a very careful and very constructive review. We concurred with all comments made, and modified the manuscript accordingly. The details of our revision follow:

Reviewer #3:
In this manuscript, the authors set out to test the effect of the smell of tears on aggression. The authors collect tears from females watching sad film, and assess the effect of smelling sad tears on the level of aggression male participants demonstrate in a monetary computer game. The authors observe a decrease in aggressive behavior when participants smell tears, relative to 'trickled saline' controls. In vitro reporter assays show that tears can activate human odorant receptors expressed in cultures cells. Finally, the authors use fMRI to identify brain areas that are active during aggressive behavior in the computer game, and whose activity is modulated by tears. The analyses of imaging data suggests that tears increase functional connectivity between the left anterior insular cortex, previously implicated in aggression, and the right amygdala and piriform cortex, involved in olfactory processing.
This manuscript extends earlier findings, including by the same lab, that sad female tears contain chemosignals that can alter male testosterone levels and emotional behavior. The use of a monetary computer game to measure aggression, and the finding that tears can activate human odorant receptors in vitro provide important new information towards understanding the relevance of human tearing and its underlying brain mechanisms. With minor revisions (see below), the manuscript is a good fit for publication in PLoS.

We thank the Referee for the kind words

1) The authors highlight the role of emotional tears, but they do not provide any evidence that the effects they observed are selectively caused by emotional tears. Can the authors use e.g. reflex tears as controls for aggressive behavior and odorant receptor signaling? If so, such experiments could provide strong additional support for their conclusions.

We find that the effects are selective for tears, but the Referee is of course correct that we do not show selectivity for emotional tears versus other types of tears, particularly reflexive tears. We have been trying to collect reflexive tears for ages, yet we have found no method to obtain sufficient quantities. We have used chemicals (we actually ordered a “Hollywood kit” that helps actors cry), airflows, intranasal poke (very painful…) and more. At best, we get very watery eyes. As noted in the methods section, our studies always include a pre-study to identify prolific cryers. These are women who generate 1ml of tears or more per session, as those are the quantities we need per participant. This is a stream of tears. We are aware of no method to generate reflexive tears in such quantities. Finally, we note that our title is “Tears Provide a Chemical Blanket Protecting from Aggression”, and not “Emotional Tears Provide…”.

2) Figure 1A-C are difficult to read and interpret. The clouds of dark and light gray dots are difficult to evaluate - can the authors provide a visualization and quantification that allows to connect experiments 1 and 2? Also, if tears are odorless, how is the reader expected to interpret variability in pleasantness, intensity, and familiarity?

Thanks for pointing this out. We changed the colors to ease the evaluation and added Supplementary Figure 2 with the mean odor ratings for all descriptors. As to the variability in the plots, this is an interesting question. We think the answer reflects two factors, one technical and one psychological. The technical is that the ratings are normalized min-max. This generates a span even if all the ratings were between 0 and 0.1. The psychological is that when people rate a null stimulus, they vary in reflection of internal states. In other words, this reflects on people, not on the stimulus. This is very evident in the ratings of the blank stimulus, that vary all over the place, even though nothing is there….

3) The in vitro odorant receptor signaling assays the authors use tend to rely on high odorant concentrations, often substantially higher than used in behavioral tests. Here, tears are used undiluted in behavioral tests, but diluted ~1:30 for odorant receptor signaling assays. The authors should comment on what they estimate are physiologically relevant concentrations of odorant receptor ligands in tears.

This is a keen observation. We have not yet identified the ligands in tears responsible for the observed behavioral effect. As a result, we cannot provide any information regarding their concentrations. However, we utilized the following reasoning when assessing the impact of tears on receptor activation:
The main criteria for the tear concentration range tested against ORs was to avoid any non-OR-specific effect on cells at too high concentrations. To define this maximum concentration, we monitored the luminescence of the Renilla luciferase that is constructively produced by cells (see method section) transfected with the empty vector negative control. We first tested a wide range of concentrations (50%, 25%, and 6.25%). Since stimuli decreased the Renilla luminescence ($P=0.0545$), meaning it has an OR-independent effect on cells even at 6.25%, we refined our concentration range from 10% to 0.0316%. The highest concentration in which stimuli did not have a non-OR-specific effect was 3.16%. Thus, we monitored OR-specific activation on tears concentrations ranging from 3.16 to 1%. This information is now added in Supplementary Figure 9 and Data file 6 of the revised manuscript.

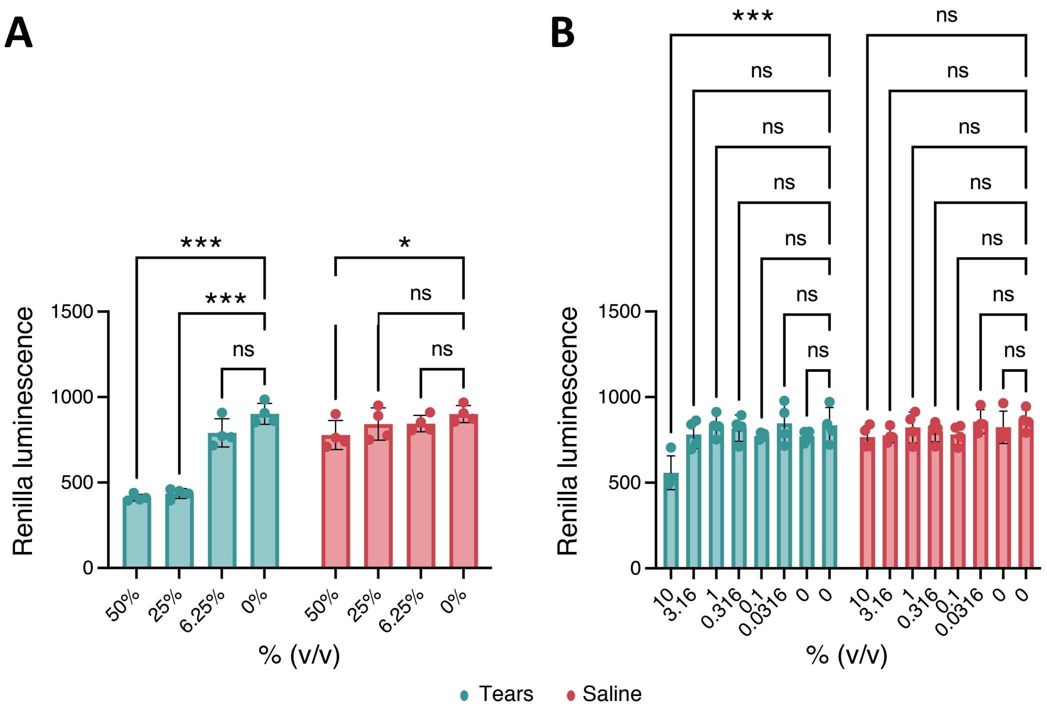

Supplementary Figure 9. **Calibration of the in-vitro assay for OR activation by tears**
We monitored Renilla luciferase luminescence produced by cells (see methods) transfected with the empty vector negative control (pCI), testing a wide range of stimuli concentrations, tears in blue and trickled saline in red. **A.** 50%, 25%, and 6.25% (v/v in CD293 stimulation medium). Tears decreased the luminescence, indicating an OR-independent effect on cells at 50% and 25% ($p < 0.001$). At 6.25% the nonspecific effect was close to significant with p = 0.0545. Therefore, we then refined the concentration range to **B.** 10% to 0.0316% (v/v). The maximum tears concentration without nonspecific OR effects was 3.16%. Statistics are done with a multiple comparison 2 way ANOVA (Dunnett test, p>0.05=ns; 0.05<p<0.01=*; 0.01<p<0.001=**; p<0.001=***).

4) For aggression behaviors during fMRI, the authors suggest that overall aggression is increased on day 2, that upon exposure to tears on day 1, this increase is amplified, and that upon exposure to tears on day 2, this increase is attenuated or eliminated. The authors should perform their statistical tests to specifically address the significance of these comparisons.

The referee is correct that, ultimately, to make this particular claim, we should conduct an experiment with two groups: one receiving saline and the other tears on both days. Then, we can observe if there is a decrease in aggressiveness on the second day only for the tears group. What we did alternatively, though it is less decisive, was to conduct an ANOVA and claim this only upon a significant interaction of condition and day. We did this, and the interaction statistic was F=3.923, p = 0.059. Although nearly significant, this remains a borderline claim, so we took it out of the manuscript altogether. We conclude that the behavioral effect in the scanner was very weak. We do not try to obscure this. In turn, we note that a one-tailed test is justified for the behavior in the scanner, as it followed the lab experiment where tears reduced

aggression, so this is the hypothesis tested. Thus, we now report in the main manuscript: "In turn, in this challenging experiment, the behavioral effect of tears on aggressive behavior was only subtle. The mean APR ± SD following trickled saline was 1.306±1.6, and APR following tears was 0.967±1.357 (Shapiro-Wilk, $W = 0.857$, $p < 0.002$, implying a non-normal distribution dictating a nonparametric test). Given the results of Experiment 1 where tears significantly reduced aggression, we apply a one-tailed hypothesis: Wilcoxon signed rank $Z = 67$, effect size (r_{rb}) = 0.42, $p = 0.048$, one-tailed. We think this weaker (effect size of 0.541 in Exp1 vs effect size of 0.42 in Exp3) result may reflect the psychological dynamics of the day-after-day MRI experiment, and we detail this in Supplementary Figure 4."

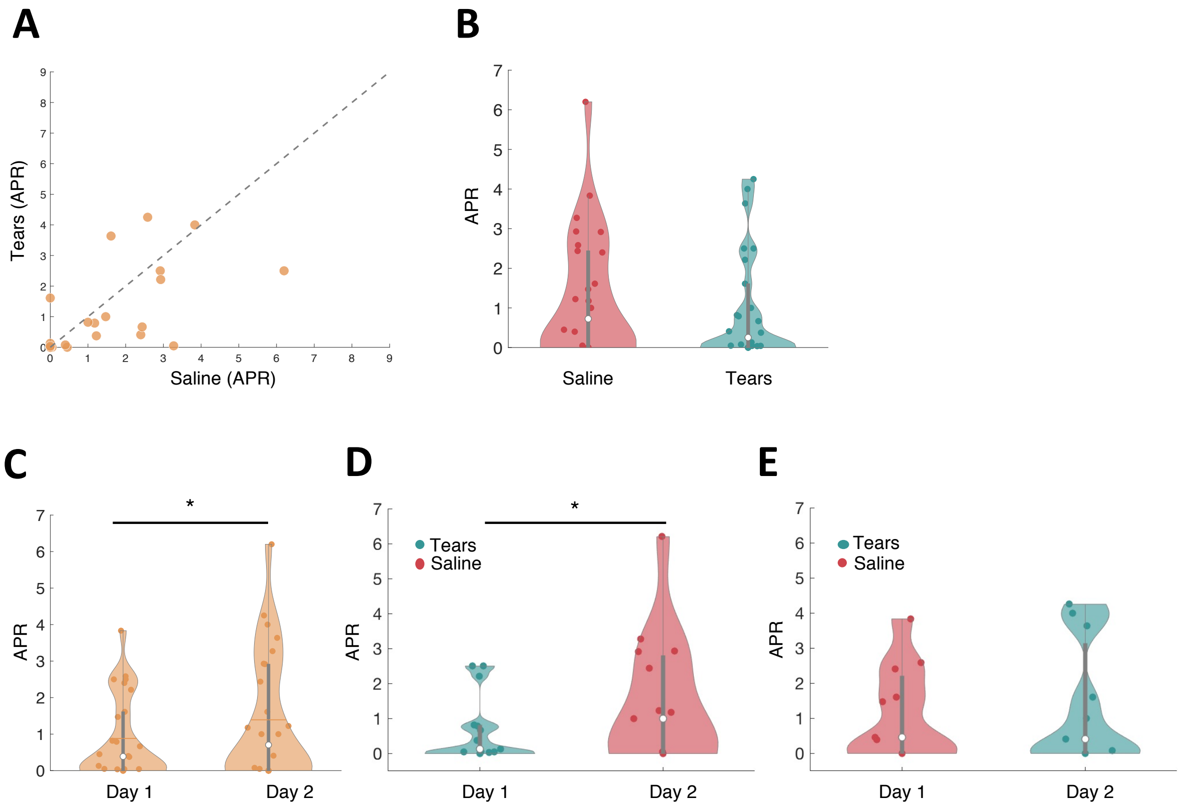

Supplementary Figure 4. The influence of tears on aggression within the MRI scanner
A. Scatter plots of the aggression ratings (APR) obtained in the MRI after exposure to tears or saline. Each dot is a participant, n=26. The data is presented along a unit slope line (X=Y), such that if points accumulate above the line, this implies higher values after tears, if points accumulate below the line, this implies higher values after saline, and if points are distributed around the line, this implies no difference. **B.** The same data as in A presented in violin-plot. Saline in red and tears in blue. The behavioral effect of tears in the MRI was more subtle, possibly since it was violated by order. Participating in a two-hour long session, almost half of it inside the MR, day after day, rendered participants significantly more aggressive on the second day, regardless of condition (mean APR difference regardless of condition (day 2 - day 1) = 0.511 ±1.2 APR, permutation $p = 0.038$, Mielke and Berry's $R = 0.125$) as depicted in **C**.

Violin plot of APR score between days, regardless of condition. Thus, participants who sniffed tears on the first day exhibited a remarkable 73.6% lower aggression under tears (mean APR tears day1 = 0.677 ± 0.942, mean APR saline day2 = 1.413 ± 1.814, Shapiro-Wilk, W = 0.704, $p < 0.001$, implying a non-normal distribution dictating a nonparametric test: Wilcoxon signed rank Z = 11, p = 0.017, effect size (r_{rb}) = 0.758. If we nevertheless use the parametric approach, the effect remains the same: t (14) = 2.34, p = 0.034, Cohen's d = 0.605), yet participants who sniffed saline on the first day had no difference in the levels of aggressiveness on the second day under tears (mean APR saline day1 = 1.159 ± 1.332, mean APR tears day2 = 1.362 ± 1.751, Wilcoxon signed rank Z = 16, p = 0.83, effect size (r_{rb}) = 0.111. If we nevertheless use the parametric approach, the effect remains the same: t (10) = 0.579, p = 0.578, Cohen's d = 0.175) as depicted in D and E respectively. **D.** Violin plot of APR by day and by stimulus when day-1 was tears. **E.** Violin plot of APR by day and by stimulus when day-1 was trickled saline. * = $p < 0.05$. Each dot in the violin plots (B-E) represents a participant. The white dot represents the median, and the grey bar represents the quartiles.

5) The manuscript, especially the introduction, appears to heavily focus on earlier work in rodents as a motivation for experiments in humans. The manuscript may benefit from a more explicit discussion of the role of human olfaction including for emotional behaviors, and its link to hormonal regulation.

We tried to focus the introduction on the signaling function of tears, and indeed, most of that research is from rodents. In consideration of the Referee comment, we have now also added discussion on the role of human olfaction in behavior, and further delve into this in the discussion.

6) What do the authors mean by 'capture a genuine link between brain and behavior'?

As the Referee clearly knows well, typical fMRI analyses rely on contrasts, and the interpretation of these can be misleading. When the results of the contrast are parametrically proportional to the manipulation, this, in our view, adds confidence. Nevertheless, given that our choice of words was unclear to the Referee, we accept that it may be equally unclear to many readers, so we have re-worded.

Reply rerefernces

1. Gračanin, A., L.M. Bylsma, and A.J. Vingerhoets, *Why only humans shed emotional tears: Evolutionary and cultural perspectives.* Human Nature, 2018. **29**: p. 104-133.
2. Vingerhoets, A.J., N. van de Ven, and Y. van der Velden, *The social impact of emotional tears.* Motivation and Emotion, 2016. **40**: p. 455-463.
3. Endevelt-Shapira, Y., et al., *Altered responses to social chemosignals in autism spectrum disorder.* Nature neuroscience, 2018. **21**(1): p. 111-119.
4. Pope, H.G., E.M. Kouri, and J.I. Hudson, *Effects of supraphysiologic doses of testosterone on mood and aggression in normal men: a randomized controlled trial.* Archives of general psychiatry, 2000. **57**(2): p. 133-140.
5. Berends, Y.R., et al., *Intranasal administration of oxytocin decreases task-related aggressive responses in healthy young males.* Psychoneuroendocrinology, 2019. **106**: p. 147-154.
6. Schlüter, T., et al., *MAOA-VNTR polymorphism modulates context-dependent dopamine release and aggressive behavior in males.* Neuroimage, 2016. **125**: p. 378-385.
