# Supplementary material for: A chemical signal in human female tears lowers aggression in males
Source: PLoS Biol. 2023 Dec 21;21(12):e3002442. doi: 10.1371/journal.pbio.3002442 (PMC10734982; doi:10.1371/journal.pbio.3002442)
Supplement: Attachment — Submitted filename: Reviewer_comments 2.pdf [file pbio.3002442.s020.pdf]

To Referee 2
We thank you for a very careful and very constructive review. We concurred with all comments made, and modified the manuscript accordingly. The details of our revision follow:

Reviewer #2:
General comments
The authors tested the influence of human tears on behavioral aggression, olfactory receptors, and fMRI activity in aggression networks. The approach is innovative and yields intriguing results in an area that is so far understudied in humans.

We thank the Referee for the kind words

However, there are some significant issues that the authors should address to increase confidence in the results. The biggest sticking point is related to experiment 3, where there are concerns about the way the behavioral results are reported, and a significant imbalance across conditions following exclusions. Other concerns are mainly related to the narrative structure of the article, and errors/inconsistencies/gaps in reporting, which are more easily addressed.

We here address constructively:

The authors have thoroughly addressed most of my comments. The main concerns I still have relate to (1) moving the behavioral results figure for Experiment 3 to the main manuscript, and (2) very large clusters emerging from the saline > tears analysis, and whether this is driven by order effects. Otherwise, my remaining concerns are all very minor. See my specific responses in blue:

Specific comments

Major comments
1. * The introduction focuses on biological mechanisms of chemical signaling (e.g., the role of ESP1), which is interesting and somewhat relevant. However, given the nature of the study, shifting the introduction's focus toward behavioral outcomes, especially in humans, would provide helpful context. For instance, the authors could briefly report behavioral and fMRI findings from Gelstein et al., 2011 (from the same group), Mishor et al., 2021 (also from the same group), and Oh et al., 2012, and discuss the known fMRI correlates of aggression.

We have modified the introduction to now also discuss the human behavioral and brain response to tears, and to manipulations of aggression. Moreover, we discuss Mishor et al and related materials in the discussion.

The authors have adequately addressed my comment.

2. * Behavioral findings from experiment 3 should be reported in a similar manner to those from experiment 1 (i.e., by comparing APR values across conditions for the entire group, and plotting the data in figures similar to those in Figure 1 [D-F]).

We concur, and have changed the relevant figures accordingly.

I appreciate the authors' transparency in addressing this comment in the manuscript text, but can they move the supplementary figure (or at least panels A-B) into the main manuscript? These could appear as new panels in Figure 1 (alongside behavioral results from Experiment 1), or in Figure 3 (alongside MRI results from Experiment 3). Also, if the authors think it's important to include their theory about order affecting behavior in the scanner, they should briefly mention it in the main text (even in the figures are relegated to the supplement). These changes could serve to curb unrealistic expectations regarding the size of this effect and/or emphasize the need to consider fMRI and ordering components for future work. Finally, can the authors adjust the statistical analysis section to account for the one-tailed nature of their new t-test?: "Normally distributed data were analyzed using ANOVAs followed by planned two-tailed t-tests, **or one-tailed t-tests when explicitly noted**."

3. Although it's plausible that order effects could be accentuated in the MRI scanning context, properly counterbalancing the conditions should account for this issue. However, based on data file 4, it seems that the groups were no longer well-balanced following exclusions, with 11 participants in the saline-first group, and 15 participants in the tears-first group. If order plays a significant role in modulating aggression as the authors suggest, this is a significant problem, since differences in fMRI correlates of aggression could be driven by the order of conditions, rather than the chemical stimulus applied. Ideally, the authors would recruit more participants to balance the groups, or at least account for the imbalance somehow in their models.

This is a keen observation with which we concur. To address this potential problem and balance the groups, we conducted the following analyses:

We have 9 fMRI participants who had saline-first. Thus, a balanced group from this perspective is reduced to 18 participants, which is borderline in power. To overcome this, we conducted two analyses. First, using a bootstrap approach, we randomly selected balanced groups of 18 participants 10,000 times, and each time conducted the analysis to create a distribution. Had we done this on the entire brain it would have taken a few months to run, therefore we limited this analysis to the two ROIs we reported in the study, namely the AIC and PFC. In a second analysis we made a one-time selection of a balanced group of 18, and conducted full brain analysis. Here we had to first decide which tears-first participants we ignore, and did this by removing the low AGQ scoring individuals to maintain two AGQ balanced groups. Both of these tests confirmed our previous findings. The bootstrap analysis revealed an average $t(17) = 2.414$, p-value = 0.037 for the left AIC, and an average $t(17) = 3.815$, p-value = 0.0015 for the PFC. New Supplementary Figure 6A-B provides additional details. The whole brain analysis of the counterbalanced group (n=18) is available in new Supplementary Figure 6C. Furthermore, the correlation between behavior and brain response in the ROIs remained consistent even in the smaller group, as shown in Supplementary Figure 6D-E.

I appreciate the authors' thorough approach in addressing my comment. In the main manuscript, the authors introduce these control analyses before bringing up that AIC and PFC emerged as significant in the initial analysis, which might be confusing for readers. I'd recommend revising the order of information for clarity. Also, could the authors implement the same color bar scale

for the supplementary figure as the main text figure for ease of comparison? Otherwise, the authors have adequately addressed my comment.

4. * Although results from this study could be related to COVID-19, this is very speculative. It's not clear that anosmia in COVID patients results in a parallel deficit in "odorless" chemical signaling, and there are a host of other (probably more salient) reasons why aggression might manifest in individuals with a complex illness. Drawing conclusions about men in particular seems inappropriate, given that there's no gender comparison in this study. Perhaps the authors could bring up the COVID-19 link, but in a more measured way, and without emphasizing it.

Agreed – removed completely

The authors have adequately addressed my comment.

Minor comments

5. * Were participants asked whether they believed their PSAP opponent was human? If not, this should not be assumed in the results section.

To ensure the integrity of the experiment's paradigm, we did not ask participants directly if they believed they were playing against a real opponent before finishing collecting the data. Instead, we collected information indirectly by asking participants about their thoughts and feelings towards their opponent, both verbally and through a questionnaire at the end of the experiment. We only excluded one participant who expressed doubt about his opponent's human identity, as social interaction is a crucial element of our study on social chemosignals. We now added further clarification in the methods section (see Questionnaires).

This makes sense. Can the authors modify the description of the paradigm to reflect this?: "In brief, in the PSAP participants play a monetary game with an opponent they **are told** is human, but is in fact a computer algorithm."

6. * It's stated that participants did not know they were sniffing tears during the sniff-jar procedure. What were the participants told, if anything, about the stimulus?

Our IRB document that participants sign, and are supposed to read before participation (although few if any read it carefully) noted that "as a participant you may receive assorted odors, including body odors, and at various intensities, above and below threshold". In other words, they generally know (and agree) that sniffing body odors is a "possibility" they may encounter in the study, but this is not up front in their attention.

The authors have adequately addressed my comment.

7. * Could the authors include bar plots to illustrate the mean pleasantness, intensity, and familiarity for tears vs. saline in Figure 1? There's also an inconsistency on the plot (which references experiments 1 and 2) and the legend and supplementary data file (which reference experiments 2 and 3) - from reading the manuscript, I believe that all of these should reference experiments 1 and 3. Most importantly though, the idea that the tears are "odorless" is not

supported by the data, since participants rate the tear stimuli as moderately intense. It would be more accurate to say that previous studies (e.g., Gelstein et al., 2011) have demonstrated that tear and saline stimuli cannot be discriminated based on odor, which implies that the chemical signal contained in tears is odorless. The data collected here support this idea (since there aren't meaningful differences in ratings across stimuli), but it is not tested directly.

Thanks for drawing our attention, we fixed this typo and added a bar plot in Supplementary Figure 2A-C. We would like to stress that the tears are perceptually odorless. The "moderate intensity" ratings applied by participants are merely because human raters rate a non-stimulus as moderate in such tests. This is clearly seen in that saline was also rated in the same way, and saline is clearly odorless. It has no VOC in it. We could have presented the data as "difference from saline", and then tear intensity would be at zero, yet we opt to show the rating data as is. Indeed, both scatter and bar plots show that tear ratings did not differ from those of trickled saline in all tested parameters, including intensity. Moreover, both stimuli (trickled saline and tears) odor descriptor ratings did not differ from blank (not trickled) saline solution (we added this information to the text and to Supplementary Figure 2D-F).

I do not think the authors have corrected the typo in the legend of Figure 1, which reads: "Each dot is the average of 10 sniffs by a given participant, light- colored dots are from Experiment 2 (n=22), and dark dots are from Experiment 3 (n=24)." Also, I'm confused as to why the odor ratings are so much higher across the board for Experiment 3 compared to Experiment 1 – is this due to the normalization approach? Finally, I take the authors' points regarding the perceptually odorless nature of tears. It's interesting that humans tend to rate non-stimuli as moderate.

8. * The APR is a ratio, but several participants have a score of 0 for one or both conditions. What does a 0 value mean in this case? And do the numbers following the mean APR (e.g., 1.67+/-1.7 APR) represent the standard error?

A score of zero means no aggressive response (0 revenge response divided by the number of provocations). The numbers following the mean APR are standard deviation (SD) and not standard error, we added this clarification to the text.

The authors have adequately addressed my comment.

9. * It does not seem appropriate to include OR1A1 in the olfactory receptor analysis, given that it does not meet the established criteria, and there are other ORs that are similarly close to the cutoff, but are not included (e.g., OR5K1). If the authors feel that it's important to include this receptor, then they should explain why in the main text (beyond stating general interest), and this should be framed as a separate exploratory analysis (after the main analysis). Could the authors also provide justification for moving forward with receptors that are activated, but not inhibited, by the stimuli (given that inhibition could also occur in a dose-dependent manner)?

We concur that retaining OR1A1 in the manuscript does not meet strict statistical inclusion criteria, and therefore we removed it. It was indeed there because we have an extended interest in it from a different study, but this is indeed irrelevant. Finally, two ORs, 2W1 and 10A4, were

inhibited by tears and not by saline in our screening assay. However, this inhibition was not confirmed by the followed dose response assay.

The authors have adequately addressed my comment.

10. * The authors state that the order of conditions was counterbalanced for experiment 1, and time of day was held constant. Can the authors clarify (in the main text) whether this was the case for experiment 3 as well?

Yes, this was the case in 3 as well, but the balance in analysis was violated following exclusions. We have now addressed this violation in Supplementary Figure 6, and find that it did not introduce or eliminate any effects.

The authors have adequately addressed my comment.

11. * The authors state: "The contrast of provocation vs. baseline regardless of condition revealed a typical salience network activation, which included typical provocation-associated regions." Framing the control condition as a "baseline" seems counterintuitive, since it implies that there's no contrast. Can the authors use another label for the control condition in the main text and associated figures to avoid confusion?

The "baseline" we refer to in this sentence is not the saline condition, it is the condition in the PSAP that is "not Provocation". We have modified the text to better clarify this.

This makes sense. Could the authors say something like this?: "**Regardless of condition, the contrast of provocation vs. non-provocation trials**…" This could be clarified in the relevant supplementary table as well.

12. * The authors should add p-value thresholds to MRI figure legends and cluster sizes to supplementary MRI tables for more complete information.

We added this information to the revised manuscript.

The authors have adequately addressed my comment. That being said, it seems strange that such large clusters emerge when comparing saline vs. tear conditions (10k+voxels in multiple areas). Is this also the case in the control analysis (where the authors chose 9 participants to appropriately counterbalance)? If not, I wonder whether this large discrepancy is due to order effects, in which case the counterbalanced analysis could be a better starting point for identifying appropriate ROIs.

13. * The authors should make it clear whether the difference metrics represented in Figure 3 B-C are subtracting the saline values from tears values, or vice versa (so the direction of the correlation is well-defined).

Thanks for the comment. This is now specified in the revised text.

The authors have adequately addressed my comment.

14. * Was the olfactory cluster the only cluster to emerge in the functional connectivity analysis? If so, this should be clarified. If not, the authors should provide a supplementary table that lists the brain regions observed (as they did for prior analyses).

Yes. This is now clarified in the text. “We observed that tears significantly impacted functional connectivity only for the left AIC, which under tears significantly increased connectivity specifically with the right temporal pole (right TP) extending into the right amygdala and piriform cortex”

The authors have adequately addressed my comment.

15. * Although results suggest that neural responses are decreased in the tear condition, and functional connectivity with an olfactory-adjacent region is increased, the authors should refrain from implying that one result causes the other (e.g., "We also find that this signal increases functional connectivity between the brain substrates of olfaction and aggression, reducing overall levels of activity in the aggression brain network.")

We did not intend to claim causality between these independent findings within our data. To stress this, we now added “we acknowledge no causal link between these findings within our data”.

The authors have adequately addressed my comment.

16. * Why were participants exposed to "blanks" (saline?) for 3 sniffs prior to the 10 main sniffs in experiment 1, and then not in experiment 2?

The added 3 “blank” sniffs are of saline that was not trickled on cheeks. This allows us to test for any perceptual differences between trickled saline and saline alone. We only need to do this once (there was no difference). Note, the three added sniffs were there for both conditions, so this does not introduce any experimental difference or imbalance. See Supplementary Figure 2D-F.

The authors have adequately addressed my comment.

17. * For the various questionnaires the participants completed, could the authors report the rationale for administering these and/or the main results? For instance, participants completed an autism quotient questionnaire - why?

In a previous study we observed significantly altered social chemosignaling in ASD [3], therefore, autism quotient is an important measure in such studies. Our participants are typically graduate students at the Weizmann Institute. We will share here with the referee that for every cohort of ~30 we run, we typically encounter at least one participant who would meet the diagnostic criteria for ASD.

This makes sense, but could the authors clarify whether they would have excluded participants who met diagnostic criteria for ASD, or whether this would simply be a factor to consider for analysis purposes?

Participants also completed anxiety and aggression questionnaires, and rated how much they would want to meet their opponent. Reporting the difference (or lack of) across conditions could be informative.

We agree that this is potentially an interesting analysis, and now ran it. We find that participants did not rate their opponent differently between conditions (Supplementary Figure 7), and there was no correlation between the participants' APR scores and their innate aggressive traits or anxiety state (new Supplementary Figure 8).

The authors have adequately addressed my comment, but note that there is a typo in the figure and legend (angary = angry).

18. * Participants also donated saliva. Why did they do this? Was the testosterone content analyzed?

Originally, we planned to measure testosterone levels in salivary samples before and after the PSAP game. The testosterone assay must be conducted for all data simultaneously at the end of the study, using the same plate, and not subject by subject. Here, in the middle of the study, we were hit by COVID, and could not analyze saliva. We ended up with samples in the freezer for a very very long time (years), and judged that it would be wrong to run assays on this material. Its still in the freezer... We now detail this in the manuscript.

The authors have adequately addressed my comment (and sorry to hear that!).

19. * Given that participants sniffed "blanks" prior to the 10 main sniffs in experiment 1, it does not seem necessary to remove the first sniff rating from the analysis. Could the authors report whether the results change if the first rating is included? There are also contradictions here, since the ratings were normalized to min-max values within all twenty odor exposures, and Figure 1 references the average of ten sniffs by a given participant.

We re-analyzed the data keeping the first sniff after the blank. Again, we observed no perceptual differences between tears and trickled saline, which did not significantly differ in perceived intensity, pleasantness, and familiarity ($F (1,20) = 1.923$, $p = 0.18$) (Figure 2A-C) (Data File 1). As for the normalization process, we applied min-max normalization to the data from both days to identify any differences between the two stimuli. This involved normalizing 20 sniffs, consisting of 10 tears and 10 saline samples. Once the normalization was complete, we used a scatter plot to compare and contrast the mean values of tears and saline. We changed the text in the manuscript for clarification.

The authors have adequately addressed my comment.

20. * For participants who were excluded for being outliers, was this based on their APR scores? Could the authors report these scores (at least in the methods and data files, and ideally in the main figure legends) for completeness? It's also unclear why a participant would be excluded for reporting that he did not believe he played against a real opponent. Again, was this probed specifically? Or based on an offhand comment? If it's the latter, it's possible that other participants felt similarly, even though they did not express it.

Yes, outliers were excluded based on their APR score. The scores of the outliers are now added to the data files (Data File 2 and Data File 4). Participants were asked to provide their overall impression of the game and write about their opponent using their own words in a post-experiment questionnaire without explicitly asking about the opponent's identity. They were also asked about it verbally by the experimenter. Though it is possible that other participants felt the same, only one indicated doubts about his opponent's human identity. Since we tested tears in the context of social chemosignals, believing in playing against a real person opponent is relevant to the context, Thus, we think it is more adequate to exclude him from the analysis [4-6]

Can the authors include this information explicitly in the manuscript when relevant (e.g., "One participant was defined as an outlier by the interquartile range **of APR scores** (< Q1-1.5 or > Q3+1.5)". Also, can the editor provide the data files so I can fully assess the authors' response?

21. * Could the authors clarify the timeline for the PASP in experiments 1 and 3? In the main text it's implied that this took 2 hours, but the authors mention a single MRI run (per day) in the methods.

For both experiments, each session lasts approximately two hours every day. In experiment 3, around 45 minutes of the session were spent in the scanner. We made it clearer in the text.

Could the authors clarify where this has been made clearer in the text? I didn't catch this, but may have missed it.

22. * Could the authors report whether there are statistically significant differences in mean time from provocation onset to monetary offset for day 1 vs. day 2, and for tears vs. saline?

There are no significant differences in the average time from provocation onset to monetary offset between days or between stimuli. This is now depicted in Supplementary Figure 10.

The authors have adequately addressed my comment.

23.* The methods reference regressing out volumes according to frame-wise displacement of greater than 0.9. Is this 0.9 mm?

Yes, we added the units to the text.

The authors have adequately addressed my comment.

24.* The authors state that they conducted whole-brain PPI analyses using three ROIs as seeds in the methods, but then only name two of these ROIs (left AIC and PFC).

This was a typo, as evident even within the original sentence: “We conducted a whole brain PPI analysis using three ROIs that emerged from the group analysis contrast of saline vs. tears as seeds to explore functional connectivity with these regions during provocation (left AIC and PFC)”. Left AIC and PFC are indeed **two** regions, and the sentence should have been: “We conducted a whole brain PPI analysis using two ROIs that emerged from the group analysis contrast of saline vs. tears as seeds to explore functional connectivity with these regions during provocation (left AIC and PFC)”. We corrected.

The authors have adequately addressed my comment.
