# Supplementary material for: A chemical signal in human female tears lowers aggression in males
Source: PLoS Biol. 2023 Dec 21;21(12):e3002442. doi: 10.1371/journal.pbio.3002442 (PMC10734982; doi:10.1371/journal.pbio.3002442)
Supplement: Attachment — Submitted filename: Agron2ndRefereeReply.pdf [file pbio.3002442.s021.pdf]

Dear Referee

We would like to once again thank you for the very careful and thorough review of our work, an effort that has allowed us to further improve on our end product. We again concurred with all comments made, and acted accordingly. Details follow, with Referee comment in red and our reply in black.

1 (related to original question 2). I appreciate the authors' transparency in addressing this comment in the manuscript text, but can they move the supplementary figure (or at least panels A-B) into the main manuscript? These could appear as new panels in Figure 1 (alongside behavioral results from Experiment 1), or in Figure 3 (alongside MRI results from Experiment 3). Also, if the authors think it's important to include their theory about order affecting behavior in the scanner, they should briefly mention it in the main text (even in the figures are relegated to the supplement). These changes could serve to curb unrealistic expectations regarding the size of this effect and/or emphasize the need to consider fMRI and ordering components for future work. Finally, can the authors adjust the statistical analysis section to account for the one-tailed nature of their new t-test?: "Normally distributed data were analyzed using ANOVAs followed by planned two-tailed t-tests, or one tailed t-tests when explicitly noted."

We complied in full. We now briefly described the order effect result in the main manuscript and adjusted the statistical analysis section as the referee suggested. We also concur that the behavioral results of Experiment 1 and Experiment 3 should be presented in the same figure. Thus, the behavioral results of both experiments are now presented in Figure 2 of the revised manuscript.

2 (related to original question 3). I appreciate the authors' thorough approach in addressing my comment. In the main manuscript, the authors introduce these control analyses before bringing up that AIC and PFC emerged as significant in the initial analysis, which might be confusing for readers. I'd recommend revising the order of information for clarity. Also, could the authors implement the same color bar scale for the supplementary figure as the main text figure for ease of comparison? Otherwise, the authors have adequately addressed my comment.

We have revised the information order in accordance with the referee's suggestion and matched the color bar scale in Supplementary Figure 6 with Figure 3.

3 (related to original question 5). This makes sense. Can the authors modify the description of the paradigm to reflect this?: "In brief, in the PSAP participants play a monetary game with an opponent they are told is human, but is in fact a computer algorithm."

The manuscript was changed accordingly.

4 (related to original question 7). I do not think the authors have corrected the typo in the legend of Figure 1, which reads: "Each dot is the average of 10 sniffs by a given participant, light- colored dots are from Experiment 2 (n=22), and dark dots are from Experiment 3 (n=24)." Also, I'm confused as to why the odor ratings are so much higher across the board for Experiment 3 compared to Experiment 1 – is this due to the normalization approach? Finally, I take the authors' points regarding the perceptually odorless nature of tears. It's interesting that humans tend to rate non-stimuli as moderate.

We have now fixed the typo, thank you.

The odor ratings in both experiments were normalized using the same approach, and there are no significant differences between the mean ratings of both experiments. The referee is correct that a stack bar plot that sums the means of both experiments is confusing. We changed the bar plot to a regular bar plot that presents the mean ratings for tears and saline from both experiments (experiments 1 and 3). This is now in Supplementary Figure 2.

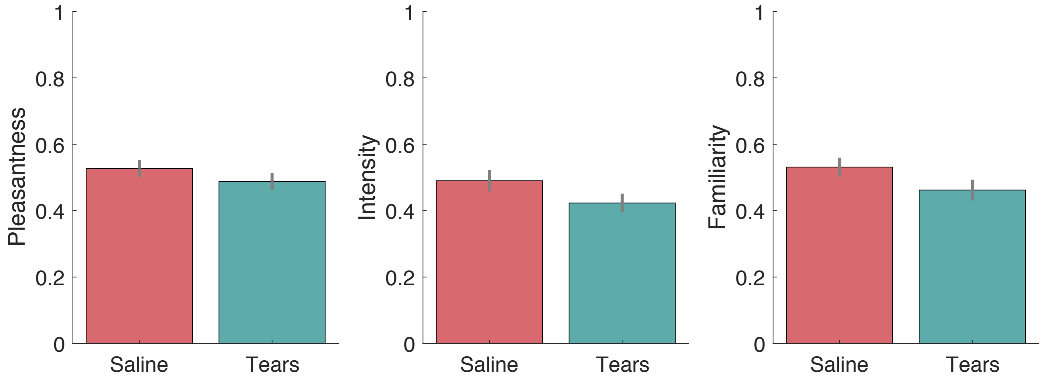

You can also appreciate the odor ratings for each experiment separately in the following bar plots:

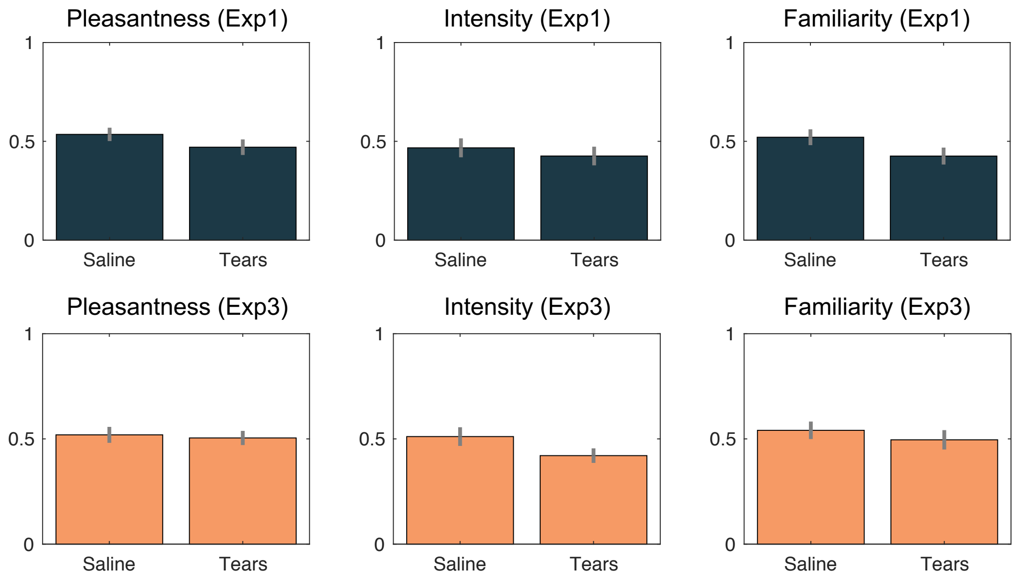

Finally, men, unlike women, tend to rate unperceived odors (Mishor et al.). This might probably stem from the default location of the VAS cursor at the middle of the VAS range.

5 (related to original question 11). This makes sense. Could the authors say something like this?: “Regardless of condition, the contrast of provocation vs. non-provocation trials…” This could be clarified in the relevant supplementary table as well.

The manuscript was changed as follows: “Provocation vs. inactive time”.

6 (related to original question 12). The authors have adequately addressed my comment. That being said, it seems strange that such large clusters emerge when comparing saline vs. tear conditions (10k+voxels in multiple areas). Is this also the case in the control analysis (where the authors chose 9 participants to appropriately counterbalance)? If not, I wonder whether this large discrepancy is due to order effects, in which case the counterbalanced analysis could be a better starting point for identifying appropriate ROIs.

The large clusters the referee mentioned are the occipital area (31176 voxels) and the right PFC (10301 voxels). In the counterbalanced control analysis, these cluster sizes are relatively similar: occipital region (20361 voxels) and right PFC (7126 voxels).

7 (related to original question 17). This makes sense, but could the authors clarify whether they would have excluded participants who met diagnostic criteria for ASD, or whether this would simply be a factor to consider for analysis purposes?

This was a factor to consider for analysis purposes, we did not exclud any participant based on their AQ score.

The authors have adequately addressed my comment, but note that there is a typo in the figure and legend (angary = angry).

Thank you, the typo was corrected.

8 (related to original question 20). Can the authors include this information explicitly in the manuscript when relevant (e.g., “One participant was defined as an outlier by the interquartile range of APR scores (< Q1-1.5 or > Q3+1.5)”. Also, can the editor provide the data files so I can fully assess the authors’ response?

The manuscript was changed accordingly. The data files were of course submitted with the manuscript.

9 (related to original question 21). Could the authors clarify where this has been made clearer in the text? I didn’t catch this, but may have missed it.

The statement on experiment duration was indeed altogether deleted in the previous revision, it is now very clearly made at the end of each “Procedures” section in the methods, where we state for Experiment 1: “All Experiment 1 procedures lasted between 90 and 120 minutes per participant per day”. And for Experiment 3: “All Experiment 3 procedures lasted around 120 minutes per participant per day, of this around 45 minutes of net scanning time”.
